# Genome-Wide Identification and Characterization of *GARP* Transcription Factor Gene Family Members Reveal Their Diverse Functions in Tea Plant (*Camellia sinensis*)

**DOI:** 10.3389/fpls.2022.947072

**Published:** 2022-06-30

**Authors:** Chuan Yue, Qianqian Chen, Juan Hu, Congcong Li, Liyong Luo, Liang Zeng

**Affiliations:** ^1^College of Food Science, Tea Research Institute, Southwest University, Chongqing, China; ^2^Chongqing Key Laboratory of Speciality Food Co-built by Sichuan and Chongqing, Southwest University, Chongqing, China; ^3^College of Life Sciences, Fujian Agriculture and Forestry University, Fuzhou, China; ^4^Key Laboratory of Tea Science in Universities of Fujian Province, College of Horticulture, Fujian Agriculture and Forestry University, Fuzhou, China

**Keywords:** tea plant, *GARP* transcription factor, expression analysis, stress response, nitrogen response

## Abstract

Golden2, ARR-B, Psr1 (GARP) proteins are plant-specific transcription factors that play vital and diverse roles in plants. However, systematic research on the *GARP* gene family in plants, including tea plant (*Camellia sinensis*), is scarce. In this study, a total of 69 *GARP* genes were identified and characterized from the tea plant genome based on the B-motif sequence signature. The *CsGARP* genes were clustered into five subfamilies: PHR1/PHL1, KAN, NIGT1/HRS1/HHO, GLK and ARR-B subfamilies. The phylogenetic relationships, gene structures, chromosomal locations, conserved motifs and regulatory *cis*-acting elements of the *CsGARP* family members were comprehensively analyzed. The expansion of *CsGARP* genes occurred via whole-genome duplication/segmental duplication, proximal duplication, and dispersed duplication under purifying selective pressure. The expression patterns of the *CsGARP* genes were systematically explored from various perspectives: in different tissues during different seasons; in different leaf color stages of tea plant; under aluminum treatment and nitrogen treatment; and in response to abiotic stresses such as cold, drought and salt and to biotic stress caused by *Acaphylla theae*. The results demonstrate that *CsGARP* family genes are ubiquitously expressed and play crucial roles in the regulation of growth and development of tea plant and the responses to environmental stimuli. Collectively, these results not only provide valuable information for further functional investigations of *CsGARP*s in tea plant but also contribute to broadening our knowledge of the functional diversity of *GARP* family genes in plants.

## Introduction

During their lifetimes, plants often encounter unfavorable or stressful conditions, including abiotic stresses such as drought, cold, salt, and nutrient deficiency and biotic stresses such as pathogen infection and herbivore attack. To respond to and resist these adverse conditions, plants have evolved several specific regulatory networks (Zhu, 2016; Zhang et al., 2021). It is well recognized that transcription factors (TFs) play fundamental roles in the regulation of plant growth and development and stress responses. In *Arabidopsis*, 2296 TF-encoding genes classified into 58 TF families have been identified and broadly investigated. Among these TF families, the Golden2, ARR-B, Psr1 (GARP) family members, which are mostly related to MYB or MYB-like TFs, compose a plant-specific TF family (Safi et al., 2017). GARP TFs contain a conserved domain named the B-motif, which is the signature motif of GARP members. However, GARPs are often mischaracterized as MYB or MYB-like TFs since their B-motifs and the MYB-like domains are highly similar. In contrast, sequence alignment showed that certain sequences of GARP and MYB-related proteins are not conserved; for example, GARP proteins contain a Ser-His-Leu-Gln-Lys/Met-Tyr/Phe (SHLQ(K/M)(Y/F)) consensus sequence, MYB-related proteins have a Ser-His-Ala-Gln-Lys-Tyr/Phe-Phe (SHAQK(Y/F)F) motif in their DNA-binding region, and all three tryptophan (W) residues are not identical between the B-motif and MYB domain (Riechmann et al., 2000; Hosoda et al., 2002; Safi et al., 2017). GARP TFs can therefore be separated from MYB or MYB-related superfamily TFs according to these specific sites. In *Arabidopsis*, 56 GARP family genes composing the phosphorus starvation response 1 (PHR1)/PHR1-like 1 (PHR1/PHL1), KANADI (KAN), NITRATE-INDUCIBLE, GARP-TYPE TRANSCRIPTIONAL REPRESSOR1/HYPERSENSITIVE TO LOW Pi-ELICITED PRIMARY ROOT SHORTENING1/HRS1 homolog protein (NIGT1/HRS1/HHO), GOLDEN2-LIKE (GLK) and type-B authentic response regulator (ARR-B) subfamilies have been identified (Riechmann et al., 2000; Safi et al., 2017); however, *GARP* family members have rarely been identified in other plant species, and their detailed information is largely unknown.

*GARP* family TFs are involved in very diverse functions in across physiological processes and stress responses in plants, such as growth and development, chloroplast development, nutrient sensing, floral transition, hormone signaling, and stress responses (Safi et al., 2017). Specifically, *GLK* genes in different species are well known to control chloroplast structure formation, and mutations in these genes cause albino leaf phenotypes and affect photosynthesis by reducing chlorophyll accumulation (Fitter et al., 2002; Powell et al., 2012; Nadakuduti et al., 2014; Chen et al., 2016; Nagatoshi et al., 2016; Lupi et al., 2019; Taketa et al., 2021). NIGT1/HRS1/HHO subgroup TFs constitute a type of NO_3_^–^-inducible TF-encoding genes in plants and function as negative regulators in the *Arabidopsis* response to nitrogen starvation by repressing the expression of other key NO_3_^–^-inducible genes, including NRT2.1 (Sawaki et al., 2013; Medici et al., 2015; Kiba et al., 2018; Maeda et al., 2018; Ueda et al., 2020b; Wang et al., 2020d). Additionally, NIGT1/HRS1/HHO TFs can bind to the promoters of inorganic phosphorus (Pi)-responsive genes, including *PHT1;1*, *PHT1;4*, and *SYG1/Pho81/XPR1* (*SPX*) family members, to directly modulate their transcription in response to Pi deficiency (Maeda et al., 2018; Ueda et al., 2020a; Wang et al., 2020d), indicating that *NIGT1/HRS1/HHO* genes play important roles in the absorption and utilization of nitrogen and Pi. Similarly, *PHR1/PHL1* subgroup members are broadly recognized to be involved in the plant response to Pi starvation and act as central regulators by regulating the transcription of Pi-responsive genes (Puga et al., 2014; Wang et al., 2014, 2018; Sun et al., 2016; Osorio et al., 2019). By functioning together with the phytohormone auxin, *KAN* has been shown to regulate organ polarity in leaves and gynoecia (Kerstetter et al., 2001; Pires et al., 2014), leaf structure (Candela et al., 2008; Zhang et al., 2009) and the shoot apical meristem (Huang et al., 2014; Xie et al., 2015; Bhatia et al., 2019; Ram et al., 2020). *ARR-B* TFs have usually been reported to function as positive regulators of cytokinin signaling and play crucial roles in plant responses to abiotic stress and developmental regulation (Argyros et al., 2008; Kim et al., 2013; Cortleven et al., 2016; Worthen et al., 2019; Wang et al., 2020a).

Tea plant (*Camellia sinensis*), a perennial evergreen woody crop species that is one of the world’s most consequential plant species, is globally cultivated in more than 50 countries (Xia et al., 2020). Recently, tea plant has gained more attention from a wide array of aspects, such as its growth and development, stress response and metabolite regulation. Many functional genes involved in the biosynthesis pathways of polyphenols, caffeine, and theanine, and in the stress response and formation of aromatic compounds have been thoroughly studied (Xia et al., 2020). Particularly, since the completion of tea plant genome sequence (Wei et al., 2018; Wang et al., 2020c), studies of tea plant genes have greatly advanced. A number of gene families whose members play critical roles in tea plant growth and development and response to stress, such as phosphate transporters (Cao et al., 2021), amino acid permeases (AAPs) (Duan et al., 2020), natural resistance-associated macrophage proteins (NRAMPs) (Li J. et al., 2021), and xyloglucan endotransglycosylases/hydrolases (XTHs) (Wu et al., 2021), and several TF family genes, such as R2R3-MYBs (Chen et al., 2021), Dofs (Yu et al., 2020), and Hsfs (Zhang et al., 2020), have been identified throughout the genome and provide a foundation for further exploring their functions in tea plant. Recently, two *CsGLK* genes were isolated from tea plant, and silencing their expression disrupted chloroplast and photosynthesis-related development in that species (Hao et al., 2020). Besides, no other *CsGARP* genes have been reported in tea plant, and little is known about the *GARP* TF family. Given that *GARP* TFs are essential in all aspects of plant developmental processes and stress responses, a thorough genome-wide investigation of tea plant is warranted.

In this study, 69 *CsGARP* TF genes were identified and characterized from the tea plant genome. Moreover, their phylogenetic relationships, gene structures, chromosomal locations, conserved motifs, gene duplication information and regulatory *cis*-acting elements were comprehensively analyzed. The subcellular localization detection showed that CsNIGT1b, CsKAN1b, and CsBOA1 were localized in cell nucleus. The expression patterns of *CsGARP* genes in different tissues during different seasons, in different leaf color stages, under aluminum (Al) treatment and nitrogen treatment, and in response to abiotic and biotic stresses were also investigated. The results provide valuable information for further functional studies of *CsGARP* genes in tea plant.

## Materials and Methods

### Collection of Sequence Data

The *Camellia sinensis* genome sequence and 18 additional representative genome sequences were used for comparative analyses. The species included 2 basal angiosperms, 4 monocots and 13 dicots (5 superasterids and 8 superrosids). The cds files and generic feature format (gff) files and the genome data for tea plant (cultivar Suchazao) were downloaded from the Tea Plant Information Archive (TPIA^1^) (Xia et al., 2019), and the data for *Actinidia chinensis* was downloaded from the Kiwifruit Genome Database (KGB^2^) (Yue et al., 2020). The protein sequences of another 17 species were downloaded from Phytozome v12.1^3^ (Goodstein et al., 2012). The species information is detailed in Supplementary Table 1.

### *GARP* Gene Family Identification and Phylogenetic and Gene Structure Analysis

According to the report by Zhu (2016), the amino acid sequences of 56 GARP gene family members in *Arabidopsis thaliana* were retrieved from The Arabidopsis Information Resource (TAIR^4^) and used to perform local BLASTP searches (*E*-value-5) to identify homologous genes in tea plant and other plant species. GARPs form a distinct group of TFs distantly related to MYB superfamily TFs, and MYB and MYB-like TFs were also included in the original BLASTP results. To separate GARP family member sequences from MYB-related sequences, phylogenetic analyses were conducted inclusive of all candidate sequences screened above and those from *Arabidopsis thaliana*. The MAFFT program (with the default parameters) was used to perform multiple sequence alignments of the GARP protein sequences, and the preliminary tree construction of the aligned GARP protein sequences was based on the maximum likelihood method and performed via FastTree software. Moreover, to further ensure the reliability of the identified GARPs, the sequences were aligned using ClustalX to determine the conserved sites of the GARP-specific B-motif in tea plant, after which all candidate sequences and those from *Arabidopsis* were confirmed by comparison with GARP member sequences through searches of the NCBI Conserved Domain database and the SMART database. Apart from those of *Arabidopsis thaliana*, the sequences of GARPs from other plant species were identified as described above.

A phylogenetic tree was also constructed via the same strategy including GARP from tea plant, *Vitis vinifera* and *Arabidopsis thaliana*, and the tree was constructed by the use of the Evolview website^5^. Based on the well-identified GARPs from *Arabidopsis thaliana*, the phylogenetic tree was divided into five branches. To visualize the gene structure of the exon–intron distribution of the *CsGARP* genes, the coding DNA sequence (CDS) of each *CsGARP* gene and its corresponding genomic DNA sequence were uploaded and analyzed using the online Gene Structure Display Server^6^.

### Chromosomal Locations of *GARP* Genes in Tea Plant

To analyze the chromosomal locations of the *CsGARP* gene family members in tea plant, the gff files and IDs of the tea plant *CsGARP* were used to map the locations of *CsGARP* genes by TBtools software (Chen et al., 2020).

### Conserved Motif Analysis

The conserved motifs of the CsGARP protein sequences in tea plant were predicted through the MEME online program^7^ with the following parameters: number of repetitions = any; maximum number of motifs = 15; and optimum motif length = 6–200 residues.

### Gene Synteny and Evolutionary Rate Analyses

*CsGARP* gene duplication information was investigated using BLASTP with filters including a percent identity > 75% and a query coverage > 75% of the query length. Gene pairs with less than 100 kb on the same chromosome and > 100 kb distance on the same or even different chromosomes were considered tandemly duplicated (TD) and segmentally duplicated (SD) gene pairs, respectively. Synonymous (Ks) and non-synonymous (Ka) substitution rates were estimated using KaKs_Calculator 2.0 (Wang et al., 2010), and the diversion year was calculated according to T = Ks/2λ, where the clock-like rate (λ) was assumed to be 6.5 synonymous substitutions per 10^–9^ years (Wang et al., 2021). Genome and protein sequence information for *Vitis vinifera*, *Arabidopsis thaliana* and *Actinidia chinensis* were downloaded from Phytozome v12.1 (see text footnote 3), TAIR (see text footnote 4), and the KGD (see text footnote 2), respectively. The synteny of *CsGARP* genes was predicted and represented using MCScanX (Wang et al., 2012), with the default parameters. The orthologous/homologous gene pairs were displayed as chord diagrams using the Perl circos package (Naquin et al., 2014).

### Analysis of Regulatory *Cis*-Acting Elements

To further analyze the regulatory mechanisms of the *CsGARP* genes in tea plant in response to stress and growth and development, the sequence of the region 1500 bp upstream of the translation start site of the *CsGARP* genes was downloaded from the TPIA database^8^, and the putative *cis*-acting elements were identified through the PlantCARE program^9^. The *cis*-acting elements involved in abiotic stress responses, biotic stress responses, tissue-specific expression, light responses, the circadian rhythm and the cell cycle, as well as core promoter elements, were summarized and analyzed.

### Expression Pattern Analysis

A total of five publicly available RNA sequencing (RNA-seq) datasets were downloaded from the Short Read Archive of the NCBI database for expression analysis in different tissues of tea plant (project accession number: PRJEB39502), at different leaf color stages (project accession number: PRJNA277458), in response to cold stress (project accession number: PRJNA411886), in response to salinity and drought stress (project accession number: PRJEB11522), and in response to Al treatment (project accession number: PRJNA517582). The sequence database of RNA in response to *Acaphylla theae* attack was maintained in our laboratory. The raw read counts for each transcript were calculated using the HISAT2 and featureCounts pipeline and then normalized to transcripts per million (TPM). A heatmap was generated and visualized using TBtools software. The color scale shown with the heatmap represents the TPM counts, and the ratios were log2 transformed.

### Nitrogen Treatment and Gene Expression Analysis via qRT–PCR

Since tea plants preferentially take up ammonium (NH_4_^+^) rather than NO_3_^–^, NH_4_^+^ treatment was employed to investigate the expression patterns of *CsGARP* genes in response to nitrogen stimuli. The expression levels of genes in tea plant were measured in response to different doses of nitrogen treatment. One-year-old tea plants (*Camellia sinensis* cv. *Longjing 43*) were grown hydroponically as previously described by Tang et al. (2020). Nutrient solutions consisting of 0, 1.5, 3, and 6 mmol (NH_4_)_2_SO_4_ were used to cultivate tea plants under controlled growth conditions of 26/22°C (light/dark: 14/10 h) air temperature, 70% relative humidity and a light intensity of 200 mmol m^–2^ s^–1^ for three weeks. Afterward, young leaves were sampled, and their total RNA was extracted using an RNAprep Pure Plant Kit (Tiangen, Beijing, China). First-strand cDNA was synthesized using a Prime-Script™ RT Reagent Kit with gDNA Eraser (TaKaRa, Dalian, China). qRT–PCR primers of 20 genes were designed with Primer-BLAST^10^ and are listed in Supplementary Table 2. qRT–PCR amplifications were performed on the LightCycler 480 II Real-Time PCR System (Roche, Switzerland) using 10 μL reaction mixtures consisting of 5 μL of SYBR qPCR Master Mix (High ROX Premixed, Vazyme, Nanjing, China), 1 μL of cDNA, 1 μL of each primer (10 mM), and 3 μL of RNase-free water, as described by Yue et al. (2019). Two technical replicates and three biological replicates were carried out for all qRT–PCR quantifications. The transcript results were calculated according to the 2^–ΔΔ*Ct*^ method, with the *CsPTB* gene serving as a housekeeping gene.

### Subcellular Localization Assay

The *CsNIGT1b*, *CsKAN1b*, and *CsBOA1* were selected to detect subcellular localizations of their encoded proteins on tobacco leaves. The open reading frames (ORFs) of these genes were amplified and inserted into the pCAMBIA2300-35Spro-GFP vector. The recombinant plasmids were transformed into *Agrobacterium* GV3101 and then transfected into the 4- to 6-week-old tobacco leaves. The infiltrated plants were grown under dark condition for 2 to 3 day and the subcellular localization were observed with the fluorescence confocal microscopy (Carl Zeiss, Germany). The primers used for ORFs cloning were listed in Supplementary Table 2.

## Results

### Identification of *CsGARP* Transcription Factors in Tea Plant

**FIGURE 1:**
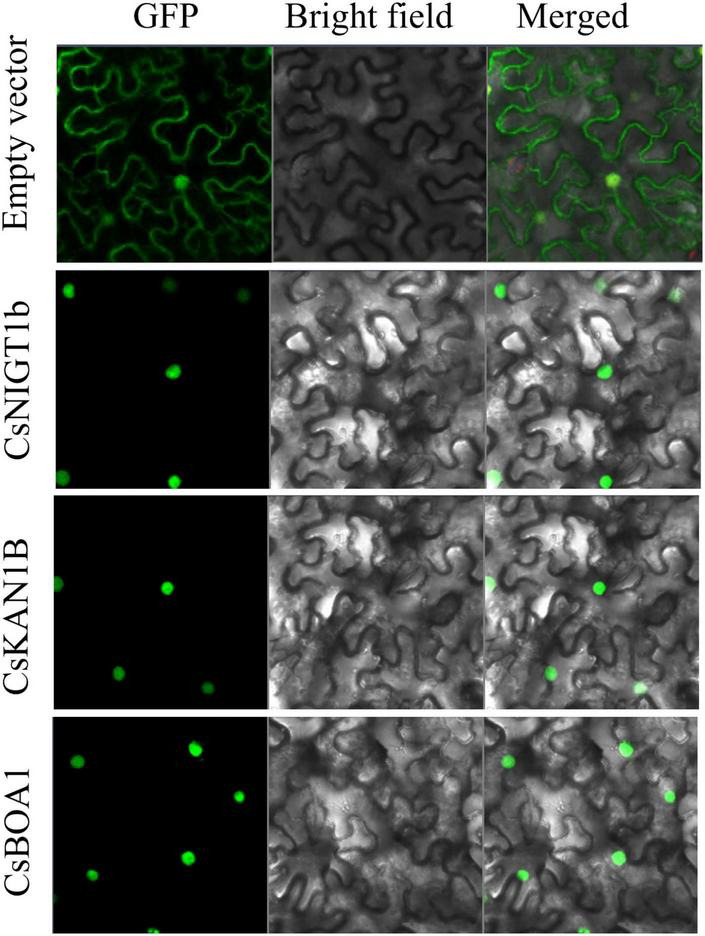
Subcellular localization of CsNIGT1b, CsKAN1b, and CsBOA1 proteins. The free GFP and recombinant GFP proteins of CsNIGT1b, CsKAN1b, and CsBOA1 were transiently expressed in tobacco leaves and observed using a fluorescence confocal microscopy.

Since GARP proteins are highly similar to MYB or MYB-like TFs in terms of both sequence and structure, candidate genes with typical identical GARP amino acids were compared and screened according to the methods of Safi et al. (2017). In total, 69 *GARP* genes were identified in tea plant after the putative MYB or MYB-like sequences were removed, and the *GARP* genes were named based on their subfamily location. The lengths of the sequences of the GARP proteins ranged from 136 (CsKAN1g) to 685 (CsARR-B4c) amino acids, and accordingly, the molecular weight varied from 15.52 kDa to 74.68 kDa. The theoretical isoelectric point (pI) ranged from 5.05 (CsPHL6b) to 10.62 (CsKAN1e). All the proteins had grand average of hydropathy (GRAVY) values below zero, suggesting that they are all hydrophilic proteins. Detailed information concerning these genes, including their name, chromosome position, instability index II value, aliphatic index value and predicted subcellular localization, is listed in Table 1. The GARP proteins were predicted to be most likely located in the nucleus, while several were mainly predicted to be located in the cytoplasm (CsARR-B1b, CsARR-B2b, CsARR-B1a, CsPHL2c and CsPHL3b), mitochondrion (CsKAN1g), nucleus and cytoplasm (CsMTF3), and chloroplast (CsKAN1e, CsPHL4b, and CsPHL1b). Additionally, *CsNIGT1b*, *CsKAN1b*, and *CsBOA1* were selected to perform subcellular localization assay; as showed in Figure 1, all proteins were localized in cell nucleus as predicted.

**TABLE 1:** Information of *CsGARP* genes identified in tea plant.

Sub-Family	Gene_ID	*CsGARP* names	Chromosome			Amino acid size	Mw (kD)	PI	GRAVY	Instability index II	Aliphatic index	Subcellular location
			Location	Start	End							
ARR-B	CSS0008648.1	*CsARR-B1b*	Contig78	165516	167706	287	32.75	6.25	−0.6	47.23	82.44	cyto,cysk,nucl
	CSS0010394.1	*CsARR-B4d*	Contig534	58324	68671	676	73.59	6.4	−0.429	52.99	79.75	nucl
	CSS0013426.1	*CsARR-B2d*	Chr10	8419100	8424797	594	66.61	6.51	−0.513	46.66	77.74	nucl
	CSS0017468.1	*CsARR-B2b*	Chr11	27269355	27273709	572	64.59	6.42	−0.456	41.91	85.17	cyto,nucl
	CSS0024071.1	*CsARR-B4a*	Chr12	27400297	27419246	667	71.89	6.74	−0.379	53.97	82.14	nucl
	CSS0024543.1	*CsARR-B3a*	Chr11	6235734	6237292	399	45.28	5.45	−0.682	51.41	73.76	nucl
	CSS0035353.1	*CsARR-B1a*	Chr6	161617767	161619797	283	32.33	6.43	−0.602	45.56	82.58	cyto,cysk,nucl
	CSS0037737.1	*CsARR-B2a*	Chr6	7338807	7343330	571	64.57	6.1	−0.474	42.13	80.37	nucl,cyto
	CSS0039789.1	*CsARR-B2c*	Chr10	9042578	9047999	594	66.61	6.51	−0.513	46.66	77.74	Nucl
	CSS0042562.1	*CsARR-B4c*	Chr6	41056441	41066639	685	74.68	6.86	−0.463	52.44	78.28	Nucl
	CSS0043810.1	*CsARR-B4b*	Contig364	2958	21280	667	71.96	6.87	−0.393	54.97	81.56	Nucl
	CSS0049738.1	*CsARR-B3b*	Chr9	6270660	6276132	678	74.58	5.72	−0.492	51.73	76.64	Nucl
	CSS0037714.2	*CsAPRR1*	Chr14	34336782	34343114	553	61.75	5.37	−0.591	54.81	71.92	nucl,cyto
GLK	CSS0026556.1	*CsMTF3*	Chr10	143873558	143874584	291	31.90	6.8	−0.312	58.14	75.12	nucl_plas, golg, nucl
	CSS0041137.1	*CsMTF1*	Chr13	40176187	40178190	292	31.59	6.8	−0.508	59.83	70.21	nucl, nucl_plas
	CSS0044249.1	*CsMTF2*	Chr10	144027289	144029380	289	31.27	6.73	−0.535	54.75	66.57	nucl, nucl_plas
	CSS0003719.1	*CsBOA1*	Chr11	66026281	66028864	317	34.90	6.32	−0.653	46.66	64.01	nucl
	CSS0034150.1	*CsPCL1*	Chr4	138908304	138910584	297	32.95	5.76	−0.644	54.6	71.55	nucl
	CSS0000687.1	*CsGLK1*	Chr7	58555200	58559618	439	47.44	6.77	−0.653	56.28	62.41	nucl
	CSS0001889.1	*CsGLK2*	Chr5	109020611	109028465	432	47.76	6.97	−0.603	60.36	72.66	nucl,chlo,cyto
	CSS0036016.1	*CsGLK3*	Chr5	108867197	108881249	439	48.48	6.59	−0.601	66.67	73.51	nucl,cyto,chlo
KAN	CSS0001412.1	*CsKAN1f*	Chr12	126646066	126651938	353	39.60	7.91	−0.838	51.3	58.61	nucl
	CSS0003450.1	*CsKAN2c*	Chr8	134932824	134935058	409	46.68	8.34	−0.916	56.37	64.55	nucl
	CSS0004133.1	*CsKAN1c*	Chr1	125927391	125948401	387	42.88	7.22	−0.739	58.09	64.03	nucl
	CSS0006502.1	*CsKAN2f*	Chr4	131665280	131667056	323	36.62	6.84	−0.799	49.72	69.72	nucl,cyto
	CSS0006880.1	*CsKAN1a*	Chr9	123807459	123810794	324	36.21	7.53	−0.69	45.67	64.69	nucl,cyto_nucl
	CSS0011420.1	*CsKAN2i*	Chr4	188988816	188990564	341	38.52	7.83	−0.931	64.31	65.78	nucl
	CSS0018275.1	*CsKAN2d*	Contig865	98336	100569	425	48.69	8.06	−0.897	54.88	63.72	nucl
	CSS0021269.1	*CsKAN1h*	Contig14	16493	22502	366	41.36	8.29	−0.886	53.68	54.37	nucl
	CSS0023102.1	*CsKAN1b*	Chr4	17185120	17189339	313	34.64	7.5	−0.599	36.54	62.62	nucl
	CSS0023934.1	*CsKAN1e*	Chr1	117052444	117054662	249	27.85	10.62	−0.608	61.22	64.62	chlo,nucl
	CSS0031744.1	*CsKAN2e*	Contig287	79856	81708	365	41.09	6.68	−1.029	52.25	63.04	nucl
	CSS0039972.1	*CsKAN1d*	Chr2	55963193	55972892	452	50.17	7.37	−0.764	58.37	61.88	nucl
	CSS0043198.1	*CsKAN2h*	Chr10	140457503	140458436	161	17.95	9.71	−0.765	66	71.37	nucl
	CSS0043572.1	*CsKAN2b*	Contig1113	259705	261760	236	27.00	6.51	−0.915	66.06	57.84	nucl,cyto_nucl
	CSS0047374.1	*CsKAN1g*	Chr14	30539257	30541051	136	15.52	10.52	−0.788	60.22	46.64	mito,nucl,cyto_nucl
	CSS0047937.1	*CsKAN2a*	Chr6	45661755	45663836	237	27.13	6.51	−0.926	66.64	57.59	nucl,cyto_nucl,mito
	CSS0049732.1	*CsKAN2g*	Chr10	140910339	140911272	161	17.95	9.71	−0.765	66	71.37	nucl
NIGT1/HRS1 /HHO	CSS0006078.1	*CsNIGT1b*	Chr10	32873850	32875167	286	32.05	6.57	−0.953	63.41	62.73	nucl
	CSS0007741.1	*CsNIGT2a*	Chr6	61166921	61169904	476	51.90	6.98	−0.781	56.9	61.16	nucl
	CSS0007827.1	*CsNIGT2c*	Chr14	95838707	95841430	457	50.28	6.86	−0.801	60.93	60.88	nucl
	CSS0014748.1	*CsNIGT2g*	Chr4	106104011	106107124	389	43.69	6.52	−0.904	71.75	60.41	nucl
	CSS0031217.1	*CsNIGT1a*	Chr6	28314264	28318894	284	32.30	9.7	−0.833	58.77	68.35	nucl,cyto_nucl
	CSS0031258.1	*CsNIGT1c*	Chr10	32618279	32621692	396	43.66	7.88	−1.044	71.77	55.23	nucl
	CSS0031273.1	*CsNIGT2b*	Chr6	60415340	60418566	476	51.99	7.07	−0.79	57.82	61.16	nucl
	CSS0044539.1	*CsNIGT2e*	Chr1	194296853	194299074	285	31.93	8.5	−0.541	45.18	82.74	nucl,cyto
	CSS0044591.1	*CsNIGT2d*	Contig1019	353441	356364	458	50.40	6.88	−0.806	60.57	60.52	nucl
	CSS0049001.1	*CsNIGT2f*	Chr3	14497450	14499730	384	42.55	8.47	−0.77	58.48	69.82	nucl
PHR1/PHL1	CSS0005408.1	*CsPHL5b*	Chr13	1851896	1854544	374	41.90	7.23	−0.86	70.24	57.43	nucl
	CSS0006111.1	*CsPHL2a*	Chr12	122342467	122346104	283	31.70	9.5	−0.714	32.15	77.84	nucl,cyto
	CSS0008911.1	*CsPHL4c*	Contig149	492337	498750	338	36.60	6.8	−0.644	50.85	72.4	nucl,chlo
	CSS0015223.1	*CsPHL6a*	Chr13	77400743	77410969	486	52.88	5.05	−0.631	59.62	67.86	nucl
	CSS0017966.1	*CsPHL5a*	Chr13	899787	902692	411	46.26	7.26	−0.854	57.71	67.68	nucl
	CSS0018618.1	*CsPHL1a*	Chr12	76523537	76525316	339	38.14	7.77	−0.847	47.33	67.58	nucl
	CSS0019219.1	*CsPHL4a*	Chr10	137618503	137623650	271	30.91	8.42	−0.62	56.01	68.45	nucl,cyto_nucl
	CSS0019363.1	*CsPHL6b*	Contig1181	174525	186979	468	51.33	5.05	−0.662	61.82	68.57	nucl
	CSS0023343.1	*CsPHL2c*	Contig1143	718099	720828	199	22.65	10.24	−0.744	48.66	77.39	cyto,pero,nucl
	CSS0023963.1	*CsPHL3a*	Chr9	30619262	30626930	295	31.91	6.86	−0.329	43.98	86.34	nucl
	CSS0026971.1	*CsPHL6c*	Chr11	87461530	87465791	478	52.28	5.4	−0.633	63.63	69.23	nucl,chlo
	CSS0027631.1	*CsPHL3b*	Contig149	507717	515150	312	34.40	6.61	−0.634	62.11	77.6	cyto,nucl
	CSS0035819.1	*CsPHL6d*	Contig653	11723	20908	478	52.38	5.4	−0.633	63.67	70.04	nucl,chlo
	CSS0038558.1	*CsPHL4b*	Contig135	464495	469690	241	26.19	9.73	−0.72	65.94	72.86	chlo,nucl
	CSS0039772.1	*CsPHL6e*	Chr7	76609036	76619955	488	54.07	5.46	−0.639	59.68	68.93	chlo,nucl
	CSS0041856.1	*CsPHL2b*	Contig995	946	4056	212	24.05	10.12	−0.701	44.32	80.47	nucl,cyto,pero
	CSS0042630.3	*CsPHL6f*	Chr1	5166865	5173987	347	38.74	5.2	−0.859	54.29	68.56	nucl
	CSS0045704.1	*CsPHL1b*	Chr6	13777227	13778889	355	40.11	8.55	−0.8	45.39	74.42	nucl,chlo
	CSS0007601.1	*CsAPL1*	Chr7	699001	701069	376	41.68	8.36	−0.71	53.48	63.59	nucl
	CSS0018792.1	*CsAPL2*	Chr7	201928	204235	375	41.59	8.36	−0.71	53.09	63.76	nucl
	CSS0036129.1	*CsMYR1*	Chr10	165597219	165600021	419	46.74	7.5	−0.74	43.91	71.67	nucl

A total of 992 *GARP* genes were also identified and compared among 19 plant species, such as *Amborella trichopoda*, *Nymphaea colorata*, *Oryza sativa*, *Sorghum bicolor*, *Eucalyptus grandis*, *Arabidopsis thaliana*, and *Vitis vinifera* (Supplementary Table 1). The numbers of *GARP* genes differed among these plant species, of which *Glycine max* contained the most abundant *GARP* genes, while the numbers in *Amborella trichopoda*, *Colin coffee* and *Ananas comosus* were less than 35. In addition, there were more genes in the *KAN*, *PHR1/PHL1* and *ARR-B* subfamilies than in the *NIGT1/HRS1/HHO* and *GLK* subfamilies.

### Phylogenetic and Gene Structure Analysis

**FIGURE 2:**
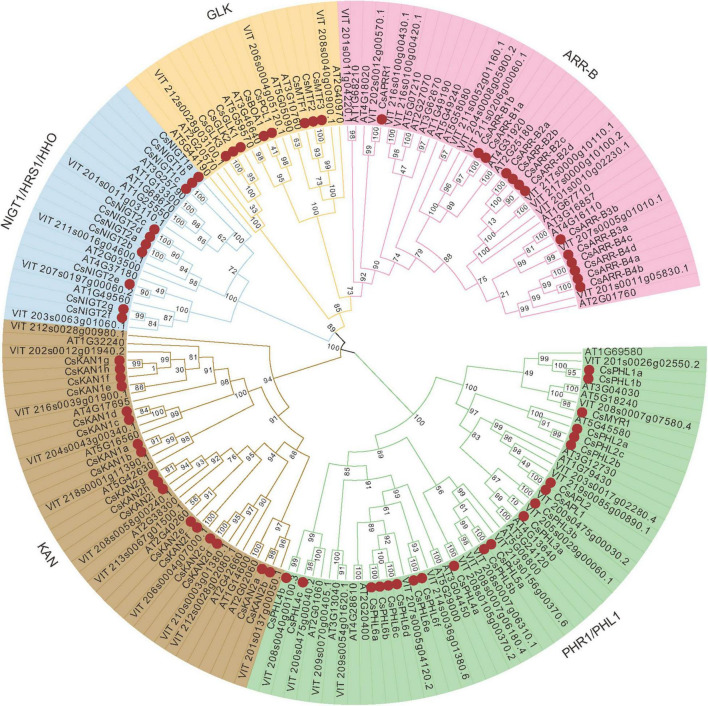
Phylogenetic tree analysis of GARP proteins from tea plant, *Arabidopsis* and *Vitis vinifera* constructed by the maximum likelihood (ML) method. The CsGARPs are marked by red dots.

To investigate the evolutionary relationships between GARPs of tea plant and GARP proteins from other species, a maximum likelihood phylogenetic tree was constructed based on the multiple alignments of the GARP amino acid sequences of 19 plant species. The GARPs could be clearly clustered into five subfamilies, i.e., PHR1/PHL1, KAN, NIGT1/HRS1/HHO, GLK and ARR-B subfamilies, as shown for *Arabidopsis* (Supplementary Figure 1). A GARP phylogenetic tree of tea plant, *Arabidopsis* and *Vitis vinifera* was also constructed and analyzed (Figure 2). In tea plant, 21 members belong to PHR1/PHL1s, 17 to KANs, 12 to ARR-Bs, 10 to NIGT1/HRS1/HHOs and 9 to GLKs. The GLK group was the smallest, while the PHR1/PHL1 group was the largest.

**FIGURE 3:**
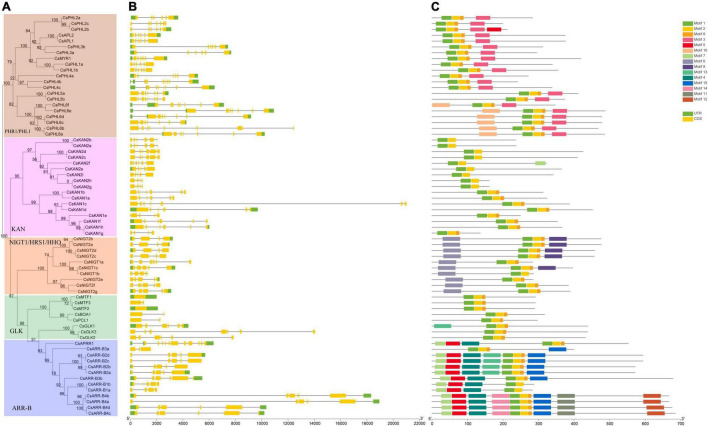
Phylogenetic relationships, gene structure and conserved protein motifs of *CsGARP* TF genes. **(A)** The phylogenetic tree was constructed using the ML method; **(B)** gene structure of *CsGARP* TF genes; **(C)** motif composition of CsGARP proteins. The motifs are colored with different boxes.

We also analyzed the gene structures of the *GARP* family members based on the tea plant genome data. As shown in Figure 3, except for *CsMTF1* and *CsMTF2*, which had no introns, all the *GARP* genes had one to 11 introns. Interestingly, *CsMTF3*, *CsBOA1* and *CsPCL1* contained one intron, and six introns were identified in *CsGLK2*, showing that *GLK* subfamily genes had high variability in terms of their gene structure. Similarly, two and 11 introns were identified in *CsARR-B3a* and *CsAPRR1*, respectively, and the rest of the *ARR-B* subfamily members had high similarity in terms of gene structure, having 4 to 6 exons, although the length of the genes was highly variable. The *NIGT1/HRS1/HHO* subfamily genes had 3 to 5 introns, and their gene structure was conserved. Although the lengths of genes in the *PHR1/PHL1* and *KAN* subfamilies were highly variable, the numbers of introns were mainly three to four and five to seven, respectively.

### Conserved Motif Analysis

**FIGURE 4:**
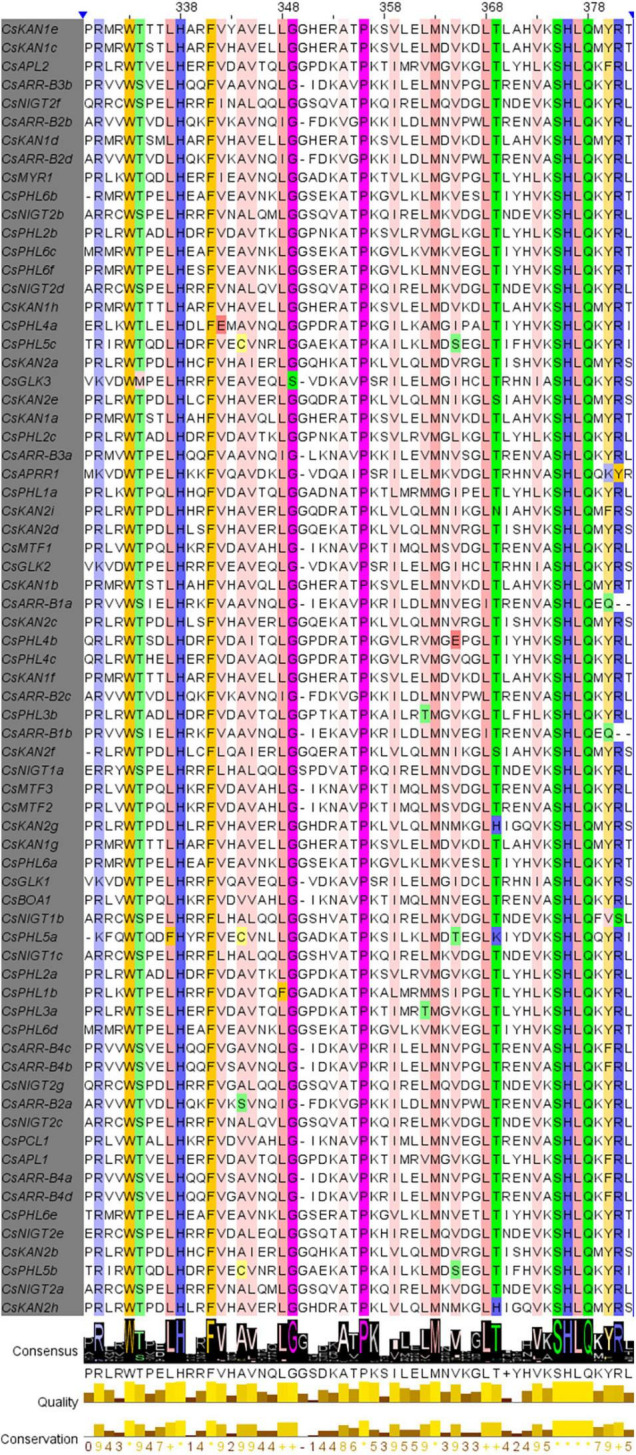
Multiple sequence alignment of the B-motif of 69 CsGARP proteins. The conserved B-motif sequences of 69 CsGARPs were determined using ClustalX, and visual analysis was performed using Jalview.

Sequence alignment of the 69 CsGARPs was conducted using ClustalX, and the results were visualized with Jalview. As shown in Supplementary Figure S2, these sequences had a low degree of conservation within the N- and C-terminal sequences, whereas they were highly conserved in the B-motif sequence, which is the signature motif of GARP proteins and is highly similar to the MYB-like domain. In particular, we observed that the sites of Trp (W), Leu-His (LH), Phe (F), Pro (P), Asn (M), and Ser-His-Leu-Gln (SHLQ) were totally conserved, and other residues, such as Arg (R), Leu-Gly-Gly (LGG), Ala (A), Lys (K), Leu (L), Thr (T) and Tyr (Y), had a high degree of similarity within the B-motif (Figure 4).

Additionally, motif analysis was conducted using MEME, and 15 types of conserved motifs were identified (Figure 3C). All the GARP proteins had motif 1 and motif 2, which referred to the B-motif of the GARP signature motif, and except for the KAN2 subgroup and CsARR-B1a/b proteins, all the rest of the proteins contained a short motif 8 after motif 2. In particular, all PHR1/PHL1 members had motif 3, motif 10 was specifically predicted to lie within the N-terminus of PHL6 subgroup members, and motif 6 was identified in all members of the NIGT1/HRS1/HHO subfamily, of which motif 9 was present within the C-terminal sequence of several proteins. Except for CsGLK1, all the GLK proteins contained only motifs 1, 2 and 8. Unexpectedly, more motif members were identified within the ARR-B subfamily members, in which motifs 4, 5, 7, 10, 11, 12, 13, 14 and 15 were present, and apart from CsARR-B3a, almost all the members had motifs 4, 5, and 7 within their N-terminal sequence, while most of them had motif 15 within their C-terminal sequence (Figure 3C).

### Chromosomal Distribution and Synteny Analysis

In total, 54 out of the 69 *GARP* genes were unevenly distributed on 14 chromosomes of the tea plant genome, while the other 15 genes were not mapped to the chromosomes. Chromosome 10 contained 10 genes, which was the most, and chromosome 6 had 8 genes. Five genes were located on chromosome 4. Chromosomes 1, 7, 11, 12, and 13 each contained 4 genes. Only one gene was mapped onto chromosomes 2, 3, and 8, and chromosome 15 had no *GARP* genes (Table 1 and Supplementary Figure S3).

**FIGURE 5:**
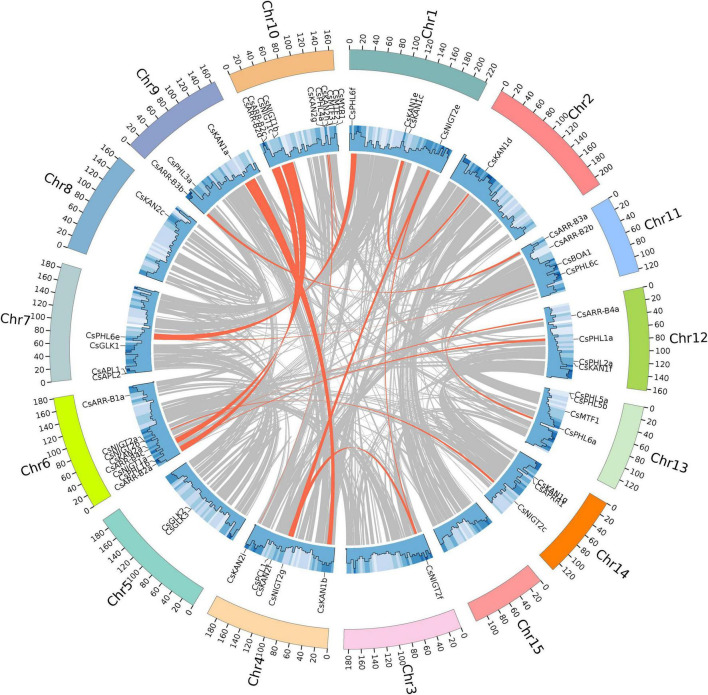
Gene location, duplication, and collinearity analysis of *CsGARPs*. The gray curves show the details of collinear regions in the tea plant (Suchazao) genome, and the red curves show the gene pairs that have undergone segmental duplication.

To analyze the evolution of the *CsGARP* TF family in tea plant, gene duplication events were analyzed. As shown in Figure 5 and Supplementary Table 3, 40 (57.97%) *CsGARP* genes were members of 23 gene pairs derived from whole-genome duplication (WGD)/segmental duplication events across chromosomes, eight genes were derived from proximal duplication, and 21 genes (30.43%) were derived from dispersed duplication. Additionally, none of the *CsGARP* genes were found to be derived from tandem duplication events. These results indicated that tea plant *CsGARP* genes were mainly generated by gene duplication during evolution. The/Ka/Ks ratio was also calculated for these duplicated gene pairs (Supplementary Table 4), all of which had a Ka/Ks ratio less than 1 (ranging from 0.23 to 0.60), suggesting purifying selective pressure occurred during *GARP* gene family evolution and a conserved function shared by these genes. Moreover, the divergence of these genes was estimated by the Ks values; the results showed that the duplication period of most of these genes was approximately 25−61 million years ago (MYA), and several genes duplication events occurred 100 MYA (Supplementary Table 4).

**FIGURE 6:**
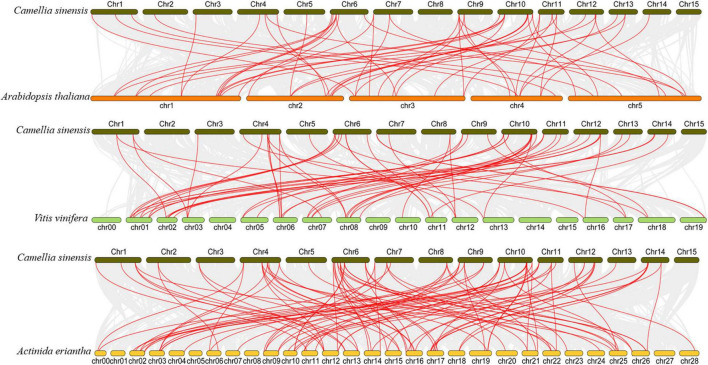
Collinearity analysis of *GARP* genes between the tea plant genome and *Arabidopsis*, *Vitis vinifera* and *Actinidia eriantha* genomes. The gray lines in the background indicate the collinearity blocks between species. The collinear gene pairs are linked with red lines.

To better understand the evolution of *GARP* genes, a comparative synteny map of *Camellia sinensis* with *Arabidopsis*, *Vitis vinifera* and *Actinidia eriantha* was constructed. As shown in Figure 6, tea plant *GARP* genes shared 53 syntenic gene pairs with *Arabidopsis* and 58 with *Vitis vinifera*. Additionally, 92 syntenic gene pairs were identified between tea plant and *Actinidia eriantha*, which constituted the largest number of background collinear blocks. Interestingly, 21 genes were found between tea plant and other plant comparative synteny maps, and these collinear gene pairs were highly conserved within several syntenic blocks, such as *CsARR-B2c*, *CsKAN2g*, *CsMYR1* and *CsNIGT1b* on Chr10 and *CsARR-B3a*, *CsBOA1* and *CsPHL6c* on Chr11, suggesting that these orthologous pairs may be important for plant evolution and that these *GARP* family genes may play fundamental roles.

### Identification of *Cis*-Acting Elements

**FIGURE 7:**
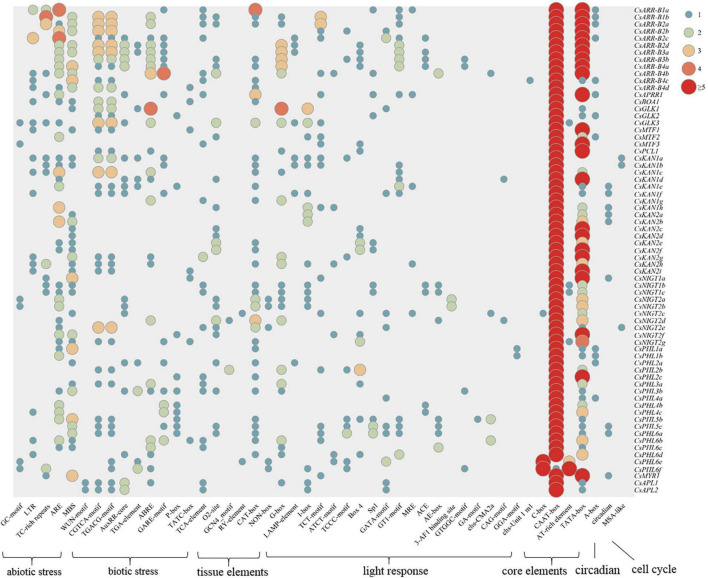
Analysis of *cis*-acting elements of 69 *CsGARP* genes. The *cis*-acting elements were classified into 7 types: abiotic stress-responsive elements, biotic stress-responsive elements, tissue-specific expression elements, light-responsive elements, core promoter elements, circadian elements and cell cycle elements.

To investigate the regulation of tea plant *GARP* gene expression, *cis*-acting elements in the 1500 bp upstream DNA sequence of the genes were identified and analyzed. As shown in Figure 7, the *cis*-acting elements in the *GARP* gene promoters could be clustered into seven classes: abiotic stress-responsive elements, biotic stress-responsive elements, tissue-specific expression elements, light-responsive elements, core promoter elements, circadian elements and cell cycle elements. All the gene promoters contained a large number of core elements, especially CAAT-boxes and TATA-boxes. Twenty-one types of light-responsive elements, such as G-boxes, B-boxes, and LAMP elements, were predicted to be present within *GARP* promoter regions. In addition, numerous stress-related elements, such as AREs, which are associated with anaerobic condition responsiveness, MBSs, which are involved in drought responsiveness, CGTCA motifs, which are related to methyl jasmonate (MeJA) responsiveness, and AREBs, which are involved in abscisic acid responsiveness, were widely distributed among the *GARP* gene members in tea plant. In particular, the stress-related *cis*-acting elements were highly clustered within the *GARP* gene promoter regions, especially in the *ARR-B* and *GLK* gene members, suggesting that *GARP* genes play critical roles in regulating abiotic and biotic stress responses. Additionally, certain elements involved in plant tissue-specific expression, such as O2_sites, GCN4 motifs, and RY-elements, were identified in the promoter of *GARP* genes, and more than half of the promoters of *GARP*s (36) contained at least one CAT-box, which is related to meristem expression, indicating that *GARP* genes are important for tea plant growth and development. Moreover, several genes had one predicted circadian-related or MSA-like element in their promoter region.

### Expression Patterns in Different Tissues During Different Seasons

**FIGURE 8:**
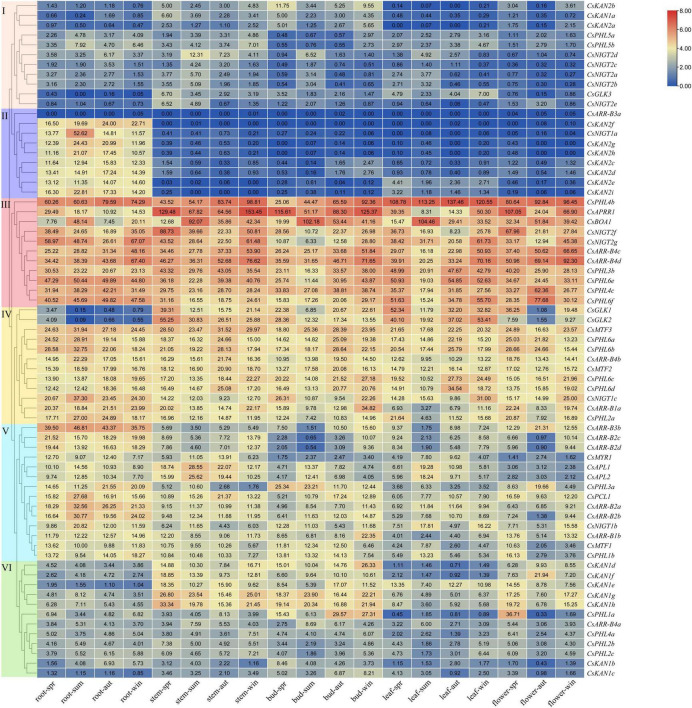
Expression analysis of *CsGARPs* in different tissues in different seasons. The *CsGARPs* were clustered into 6 groups with different colors according to their expression levels calculated with TPM values. The red and blue colors represent high and low expression levels, respectively.

The expression patterns of *GARP* genes in the roots, leaves, stems, buds and flower tissues at different seasons across years were determined and analyzed based on tea plant RNA-seq data (Wang et al., 2020c). A heatmap was generated and showed that these genes could be clustered into six groups according to their expression profiles (Figure 8). The genes of group 1 (11 genes), which were mainly *KAN* and *NIGT1/HRS1/HHO* subfamily genes, had low expression levels among the tested tissues. Similarly, the genes clustered into group 2 had low or undetectable transcript levels in the leaf, stem, bud and flower tissues, but they exhibited high levels in the roots, suggesting that these genes, especially those of several *KAN* subfamily members, had strong root-specific expression. On the other hand, several genes of group 6 had lower expression levels in the roots than in other tissues. Interestingly, 38 genes belonging to groups 3, 4 and 5, especially the group 3 genes, were highly expressed in all of these tissues. For instance, five *PHR1/PHL1* subfamily genes in group 3, i.e., *CsPHL4b*, *CsPHL3b*, *CsPHL4c*, *CsPHL6e* and *CsPHL6f*, presented high transcript abundance in the roots, leaves, stems, buds and flowers, implying that they might play critical roles in tea plant growth and development. Interestingly, we found that the expression of *CsGLK1* and *CsGLK2* was lower in the roots than in the other tissues and that a small group of *KAN* genes (*CsKAN1d/e/f/g/h*) had relatively high expression levels in the stems and buds.

Moreover, the tissue expression patterns of target genes in the spring, summer, autumn and winter seasons were investigated, and their patterns fluctuated with the seasons. For instance, in all the tested tissues, *CsAPRR1*, *CsGLK1*, *CsGLK2*, *CsGLK3* and *CsNIGT2f* had high transcript levels in the spring; *CsAPL1/WDY1*, *CsAPL2/WDY2* and *CsBOA1* had high transcript levels in the summer; *CsPHL6e*, *CsPHL6f* and *CsMTF2* had high transcript levels in autumn; and *CsARR-B1a*, *CsARR-B2c*, *CsARR-B4c*, *CsARR-B4d* and *CsNIGT2g* had high transcript levels in the winter. *CsPHL4b* was expressed at high levels in both autumn and winter.

### Expression Patterns in Different Leaf Color Stages of Tea Plant

**FIGURE 9:**
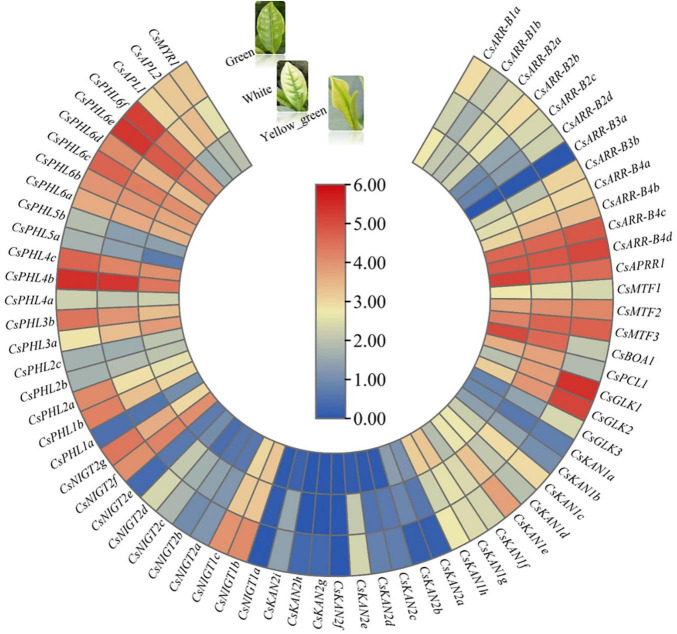
Expression analysis of *CsGARPs* in different tea plant leaf color stages. The expression levels of *CsGARPs* were calculated with TPM values. The red and blue colors represent high and low expression levels, respectively.

We investigated the expression patterns in the white-, yellow–green and green-leaf stages during tea plant young leaf development based on RNA-seq data obtained from a unique albino cultivar named Anjibaicha. More than half of the genes had low or undetectable transcript levels among these three test samples, and most of the gene expression levels were higher in the green stage than in the white and yellow-green stages (Figure 9). In particular, we found that several genes, such as *CsGLK1*, *CsGLK2*, *CsPHL6f*, *CsPHL6d*, and *CsNIGT1b*, were significantly upregulated in the green stage but were expressed at low levels in the white and yellow-green stages, suggesting that these genes are important for tea plant leaf color development. On the other hand, ten genes, including *CsBOA1*, *CsPCL1* and *CsAPRR1*, presented higher expression levels in the white and/or yellow-green stages than in the green stage; this was especially the case for *CsBOA1*, whose expression was highest in the yellow-green stage followed by the white stage, indicating that this gene might be involved in leaf color changes from the white stage to the green stage. The changes in *GARP* gene expression levels in different leaf color stages implied that these genes may function in regulating the transformation of tea leaves from being white to becoming green in color.

### Expression Patterns During Cold Stress

**FIGURE 10:**
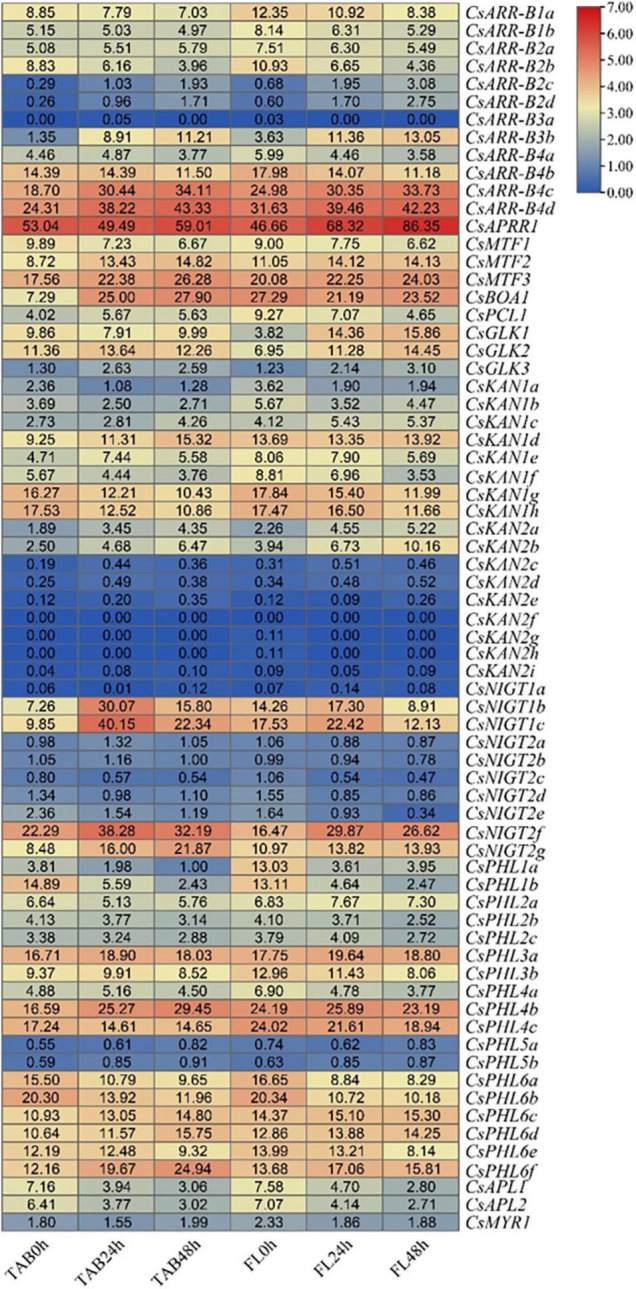
Expression analysis of *CsGARPs* in response to cold stress in FL and TAB tissues. The expression levels of *CsGARPs* were calculated with TPM values. The red and blue colors represent high and low expression levels, respectively.

We investigated and compared the expression patterns of *GARP* genes in response to cold stress between the fish leaf (FL) and the two-leaf-and-one-bud (TAB) stages (Hao et al., 2018). As shown in Figure 10, several genes, including *CsAPRR*, *CsARR-B1b*, *CsBOA1*, *CsGLK1*, *CsGLK2*, *CsKAN1d*, *CsKAN1e*, *CsNIGT2d*, *CsNIGT1b*, *CsNIGT1c*, *CsPCL1*, *CsPHL2b*, and *CsPHL4a*, exhibited different patterns between the FL and TAB stages upon cold treatment, suggesting that these genes may be associated with the difference in responsiveness of tissues under cold stress; the rest of the genes exhibited a similar tendency in response to cold stress. Interestingly, we found that certain *ARR-B* (5), *GLK* (2), *KAN* (5), *NIGT1/HRS1/HHO* (2), and *PHR1/PHL1* (2) subfamily genes were considerably upregulated, whereas most differentially expressed *PHR1/PHL1* subfamily genes were downregulated.

### Expression Patterns in Response to Salinity and Drought Stress

**FIGURE 11:**
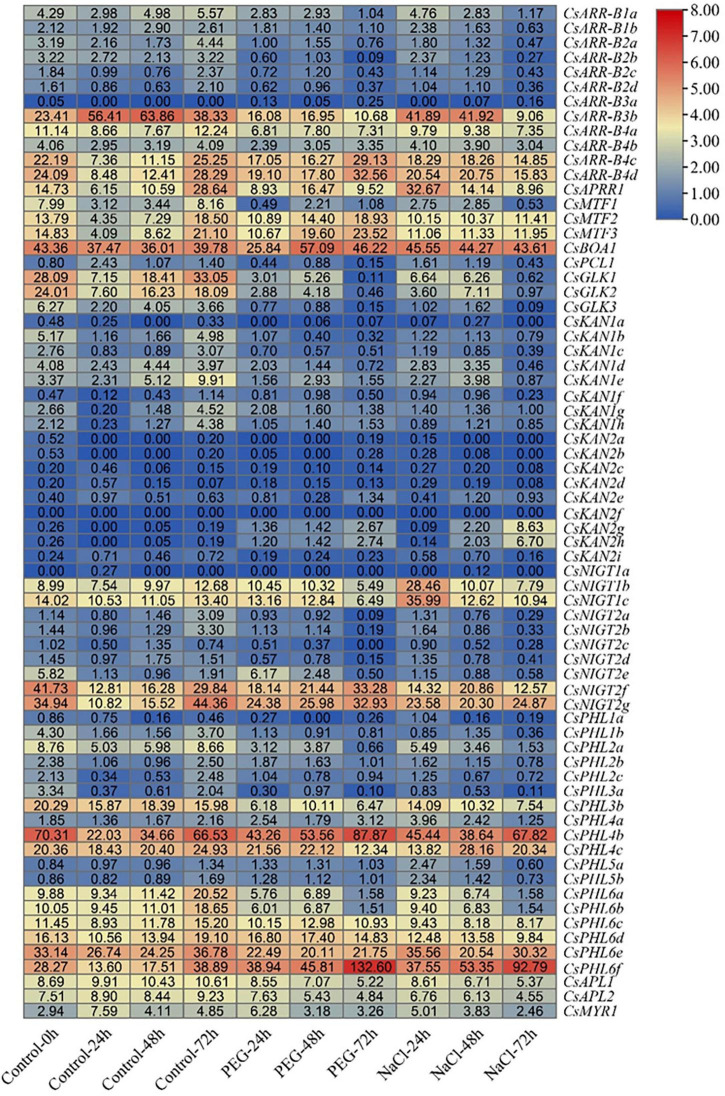
Expression analysis of *CsGARPs* in response to drought and salt stress treatments. The expression levels of *CsGARPs* were calculated with TPM values. The red and blue colors represent high and low expression levels, respectively. N: control conditions, PEG-N: drought stress imposed via 25% polyethylene glycol (PEG)6000, NaCl-N: salt stress imposed via 200 mM NaCl.

To evaluate the putative roles of *GARP* genes in the tea plant response to drought and salt stress, transcriptome data collected during continuous salinity and drought stress were isolated and analyzed (Figure 11). Most *CsKAN* subfamily member genes (11/17) had low or undetectable transcript levels among the tested samples. However, both *CsKAN2h* and *CsKAN2g* were dramatically upregulated under drought and salt treatment, whereas the expression of *CsKAN1b*, *CsKAN1c*, *CsKAN1d* and *CsKAN1e* was repressed. Interestingly, we found that the expression of *CsGLK1*, *CsGLK2*, *CsGLK3*, *CsPCL1*, and *CsMTF1* was significantly downregulated in response to drought and/or salt stress, whereas *CsMTF2, CsMTF3* and *CsAPRR1* displayed upregulation expression patterns at several points during the stress treatment. In addition, most *ARR-B* subfamily member genes, such as *CsARR-B2b*, *CsARR-B2c* and *CsARR-B3b*, were downregulated under stress, while *CsARR-B4c* and *CsARR-B4d* were induced by stress. Notably, several of *NIGT* subfamily member genes, including *CsNIGT1b*, *CsNIGT1c*, *CsNIGT2g* and *CsNIGT2f*, were upregulated in response to drought and salt treatments, while the expression of *CsNIGT2a-e* was suppressed. In addition, the expression levels of most genes in the *PHR1/PHL1* subfamily, such as *CsPHL1b*, *CsPHL3b* and *CsPHL6b*, were downregulated under drought and salt stress, while certain members—especially *CsPHL6f*—were considerably upregulated.

### Expression Patterns Under AL Treatment

**FIGURE 12:**
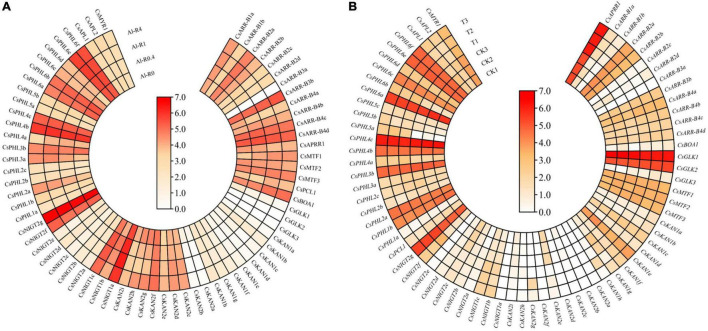
Expression analysis of *CsGARPs* under Al treatment **(A)** and *Acaphylla theae* attack **(B)**. The expression levels of *CsGARPs* were calculated with TPM values. The red and blue colors represent high and low expression levels, respectively.

Tea plant is well recognized as having high Al tolerance. In this study, the expression patterns of *GARP* genes in the roots of tea plant treated with different Al doses (0, 0.4, 1 and 4 mmol L^–1^) were analyzed (Figure 12A). With an increase in Al concentration, 19 and 24 genes were downregulated and upregulated, respectively, and the expression of the remaining genes was not affected by Al treatment. For instance, the expression levels of *CsAPRR1*, *CsGLK1*, *CsKAN2a*, and *CsNIGT2b* were gradually downregulated with an increasing Al dose from 0 to 4 mmol L^–1^, and the transcript levels of *CsARR-B3b*, *CsBOA1*, *CsKAN2d* and *CsKAN2e* increased, suggesting that the expression of these genes somewhat depended on the Al dose. Additionally, we found that the expression of *CsNIGT2g* and *CsPHL4b* was high in the tested root tissues, but the expression of these genes did not significantly change under Al treatment, indicating that these genes highly expressed in the roots may play important roles in tea plant root growth and development.

### Expression Patterns in Response to *Acaphylla theae* Attack

We investigated the expression patterns of *GARP* genes in response to *Acaphylla theae* attack. As shown in Figure 12B, the expression of most genes in leaves in response to *Acaphylla theae* infestation was not affected compared with that in healthy leaves. The expression levels of *CsKAN1h*, *CsNIGT1c*, *CsNIGT2f*, and *CsPHL6b* were significantly upregulated after *Acaphylla theae* attack, whereas the transcript levels of *CsKAN2e* and *CsNIGT2c* strongly decreased, suggesting that these differentially expressed genes might be involved in the tea plant response to biotic stress, such as attack from *Acaphylla theae*.

### Gene Expression via qRT–PCR in Response to Nitrogen Stimuli

**FIGURE 13:**
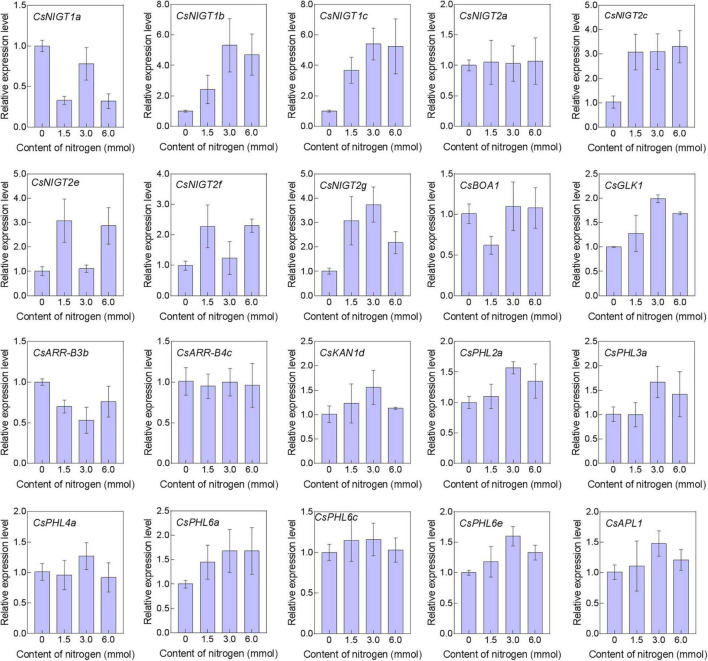
Expression analysis of *CsGARPs* under nitrogen treatment. The relative expression levels of *CsGP* genes were determined using qRT–PCR, and the results were calculated using the 2^–ΔΔ*Ct*^ method and normalized to those of the *CsPTB* housekeeping control gene.

Tea plants preferentially take up NH_4_^+^ rather than NO_3_^–^. Using qRT–PCR, we further explored the expression patterns of 20 *CsGARP* genes in response to NH_4_^+^ treatment. As shown in Figure 13, the expression of six out of eight *NIGT1/HRS1/HHO* members (*CsNIGT1b*, *CsNIGT1c*, *CsNIGT2c*, *CsNIGT2e*, *CsNIGT2f*, and *CsNIGT2g*) significantly increased under NH_4_^+^ treatment, while the transcription of *CsNIGT1a* was repressed, and NH_4_^+^ treatment did not affect the expression of *CsNIGT2a*. After NH_4_^+^ treatment, the expression level of *CsGLK1* also significantly increased, whereas the low NH_4_^+^ condition repressed *CsBOA1* transcription. The expression levels of *CsARR-B4c* and *CsKAN1d* were not affected by NH_4_^+^ treatment. Additionally, the expression of *CsPHR1/PHL1* genes was upregulated under certain treatments, but the relative expression level fold-changes were less than 2.0. These findings could provide putative gene candidates for the functional characterization and improvement of nitrogen absorption and utilization in future tea plant breeding programs.

## Discussion

The members of the GARP TF family play crucial roles in various physiological processes and stress responses in plants. According to the phylogenetic tree and conserved domain analysis, GARP TFs can be classified into *PHR1/PHL1*, *KAN*, *ARR-B*, *NIGT1/HRS1/HHO* and *GLK* subfamilies (Safi et al., 2017). GARP TFs contain a signature conserved sequence called the B-motif, which is highly similar to the MYB-like domain (Hosoda et al., 2002; Safi et al., 2017); hence, GARP TFs have been somewhat inaccurately categorized as MYB or MYB-like superfamily TFs in previous reports. The functions of *GARP* TF genes have been well elucidated in model plant species and other plant species, and 56 *GARP* members have been identified in *Arabidopsis*; however, a systematic genome-wide identification and analysis of *GARP* TFs in plants is currently lacking. In recent years, several subfamily members, such as *ARR-B*s in peach (Zeng et al., 2017) and tomato (Wang et al., 2020a) and *GLK*s in rice (Bhutia et al., 2020), have been identified and investigated across the genome; nonetheless, there have been no comprehensive investigations of the *GARP* gene family, and the functions of the *GARPs* are largely unknown in plants. In this study, we performed a genome-wide identification of the *CsGARP* gene family based on the feature sites of the B-motif that differed from those of the MYB-like domain. Sixty-nine *CsGARP* genes were identified in tea plant (Table 1), and their gene structure, chromosome location, phylogenetic relationships, conserved domains, *cis*-acting elements and tissue-specific expression patterns under various treatments were investigated.

We constructed a phylogenetic tree to perform a systematic analysis of *GARP* family genes in plants, which included tea plant and 18 other plant species. A total of 992 GARP proteins composed the tree and clustered into five subtrees, which is consistent with results for *Arabidopsis* (Safi et al., 2017), suggesting that the *GARP* TF family is ubiquitous in plants. Among these selected species, tea plant contains more *GARP* TFs than most other plant species do. Although tea plant has the largest genome size (3.1 Gb) among the species composing the tree, the number of *GARP* genes in tea plant was lower than the number in *Glycine max*, *Actinidia chinensis* and *Populus trichocarpa*, whose genome size was 0.994 Gb, 0.604 Gb and 0.434 Gb (Supplementary Table 1), respectively, indicating that the *GARP* numbers had no apparent relationship with genome size in plants. Generally, the numbers of *GARP* genes are mainly increased in certain subfamilies controlling developmental processes and the response to Pi, indicating that these pathways are important for plant evolution. We also found that the orthologous *GARP* genes between tea plant and *Arabidopsis thaliana*, between tea plant and *Actinidia chinensis*, and between tea plant and *Vitis vinifera* were primarily clustered into several syntenic blocks, such as on chromosome 4, chromosome 6, chromosome 10, and chromosome 11 of tea plant, and tea plant and *Actinidia eriantha* shared 92 syntenic gene pair relationships within the *GARP* family, indicating that tea plant and *Actinidia eriantha* are closely evolutionarily related. Wei et al. (2018) indicated that tea plant experienced two WGD events after the core-eudicot whole-genome triplication-γ (WGT-γ) event, which was shared one time with *Actinidia chinensis*. Recently, Wang et al. (2021) showed that tea plant experienced a Polemonioids-Primuloids-core Ericales (PPC)-WGD (∼63 MYA) event, which resulted in the expansion of genes involved in flavor compound biosynthesis and the stress response. Among the *CsGARP* TF family member genes, 40 out of 69 mainly resulted from segmental duplication events, 21 were derived from dispersed duplication events, and eight genes were derived from proximal duplication events (Figure 5 and Supplementary Table 3). We also estimated the divergence years of *CsGARP* genes and found that most of the genes originated approximately 25−61 MYA, and some originated approximately 100 MYA (Supplementary Table 4). Although the WGD events and the exact timepoint at which they occur in tea plant are inconclusive, most *CsGARP* genes had duplicated around the time of the most recent WGD event. Furthermore, Ka/Ks analysis showed that all of the *CsGARP* genes were under purifying selection, indicating that gene duplication events were exposed to strong purifying constraints during evolution and that duplication events, except for tandem duplications, are the main driving force in the expansion of *CsGARP* genes in tea plant.

Although GARP TFs are usually clustered into the MYB superfamily and share a MYB-like domain, the functional domain of the B-motif is distinctly different from that of the MYB domain. Safi et al. (2017) indicated that, unlike MYB proteins, GARP TFs contain only the first of three regularly spaced tryptophan residues (W) and have a consensus amino acid sequence SHLQ (K/M) (Y/F), while MYB TFs instead have the conserved sequence SHAQK(Y/F)F. These differences are present within the functional sites, implying that GARP TFs do not belong to the MYB superfamily and that GARP TFs can be separated from the MYB superfamily TFs according to the phylogenetic tree and functional domain analysis. All CsGARP proteins were predicted to contain motif 1 and motif 2, which are components of the B-motif and are important for DNA binding. In addition, different subfamily members had specific motifs. CsPHR1/PHL1 TFs have a motif 3 consistent with a coiled-coil domain, which is involved in protein–protein interactions, after the MYB-related domain; hence, these subfamily members were usually called MYB-CC family members in several previous studies (Sun et al., 2016; Wang et al., 2018). CsNIGT1 subfamily TFs contain motif 6 at the N-terminus, which is rich in hydrophobic amino acids and named the hydrophobic and globular domain (HGD), the function of which is unknown, and several members contain motif 9, which is predicted to be an EAR-like motif critical for inhibiting gene expression (Wang et al., 2020b; Li Q. et al., 2021). Among these five subfamilies, ARR-B proteins have more, unique motifs (Figure 3), implying that they may have diverse functions. Except for the MYB-like DNA-binding domain, ARR-B proteins contain a key functional receiver (REC) domain at their N-terminus, which functions as a phosphorylation-mediated switch involved in regulation and plays important roles in cytokinin-mediated plant growth and development (Hosoda et al., 2002; Cho et al., 2016). On the other hand, although the lengths of *CsGARP* genes are different, the structures of genes in the same subfamily are conserved, and each subfamily can also be classified into different subgroups. Therefore, the consensus motifs and structures of genes in each subfamily are strongly associated with the results of the subfamily classification, and the different and unique conserved motifs and sites between GARP proteins and MYB-like proteins show that GARP and MYB TFs should not be clustered into MYB superfamily TFs.

In tea plant, *CsKAN2* genes were mainly transcribed in the roots, while *CsKAN1s* had higher levels in the stems, buds and flowers (Figure 8), indicating that *CsKAN1* and *CsKAN2* may play different roles among these tissues. *KAN1* is a transcriptional repressor and plays key roles in the regulation of abaxial identity, leaf development, and meristem formation by mediating the auxin response in plants (Kerstetter et al., 2001; Candela et al., 2008; Huang et al., 2014; Xie et al., 2015; Bhatia et al., 2019; Ram et al., 2020). Recently, we observed that several *CsKAN1* genes were significantly upregulated in zigzag-shaped stems compared with normal stems (Cao et al., 2020). High transcription of *CsKAN1s* in the stems and buds during the four seasons showed that *KAN1* genes are involved in stem formation and bud development. Additionally, by interacting with *ULTRAPETALA* trxG genes, *KAN1* also regulates gynoecium patterning in *Arabidopsis* (Pires et al., 2014). *CsKAN1s* exhibited relatively high transcription in the flowers, showing that gynoecium development in tea plant is also controlled by *CsKAN1s* genes. Given that *KAN1* and *KAN2* have partially similar functions, the regulatory functions of *KAN2* are not clear. Most *CsKAN2s* genes were predominantly expressed in tea plant roots, and previous reports showed that *KAN* genes play crucial roles in lateral root development (Hawker and Bowman, 2004). Considering that *KAN* genes play critical functions in the plant auxin response, we speculate that *CsKAN2s* genes may be essential for root development through integration with the auxin pathway. On the other hand, we found that the expression levels of certain *CsKAN2s* genes, such as *CsKAN2a/b/c/d*, were upregulated in tea plant leaves in response to cold stress (Figure 10), that *CsKAN2g/h* was induced upon drought and salt stresses (Figure 11), that *CsKAN2a/b/g/h* was repressed and *CsKAN2c/d* was upregulated in response to Al treatment, and that *CsKAN2e* was downregulated in response to biotic stress, indicating that these genes might also play important roles in the tea plant response to stress stimuli.

It has been well recognized that *NIGT1/HRS1/HHO* and *PHR1/PHL1* are key regulatory TFs involved in the plant response to nitrogen and Pi. In this study, we found that most *CsNIGT* and *CsPHR1/PHL1* genes were specifically or primarily expressed in the roots (Figure 8). In *Arabidopsis*, *NIGT1* genes (*HRS1* and *HHO1*) are expressed in the transition domain of the root apical meristem and in the elongation zone (Medici et al., 2015), and *PHR1/PHL1*-related genes are primarily expressed in the roots and shoots (Rubio et al., 2001; Sun et al., 2016). These tissues play important roles in the plant response to nutrients, including nitrogen and Pi. *NIGT1/HRS1/HHO* TFs have been widely reported to be induced by NO_3_^–^. Tea plant is a high nitrogen- and Pi-consuming plant species and is considered to preferentially take up NH_4_^+^ (Tang et al., 2020). In this study, we determined the expression patterns of 20 *CsGARP* genes, i.e., 8 *CsNIGT1/HRS1/HHO* genes, 7 *CsPHR1/PHL1* genes, 2 *CsGLK* genes, 2 *CsARR-B* genes and *CsKAN1d* gene, in response to NH_4_^+^ treatment (Figure 13). Interestingly, although *NIGT1/HRS1/HHO* genes have been characterized as NO_3_^–^-induced genes (Sawaki et al., 2013; Maeda et al., 2018), 6 out of 8 *CsNIGT1/HRS1/HHO* genes were significantly upregulated in response to NH_4_^+^ treatment, indicating that *CsNIGT1s* are also involved in the tea plant response to NH_4_^+^ treatment and might play important roles in nitrogen absorption and utilization in tea plant. Recently, numerous differentially expressed genes were identified via transcriptome sequencing in tea plant in response to nitrogen treatment (Li et al., 2017; Ruan et al., 2021), and our results could provide valuable candidate genes for further studies on the transcriptional regulatory mechanism of the tea plant response to nutrients. Additionally, in this study, we found that the expression levels of several *CsNIGT1/HRS1/HHO* and *CsPHR1/PHL1* genes were also regulated by stress treatments. Environmental stresses, including drought, cold and salt, impact plant growth and physiological processes by modulating nutrient metabolism, such as carbon nitrogen and Pi metabolism (Ding et al., 2018). Recently, the occurrence of cross-talk among nutrient utilization, normal growth and development and stress response has been well established (Wu et al., 2020; Han et al., 2021; Sevanthi et al., 2021), yet the putative functions of *NIGT1/HRS1/HHO* and *PHR1/PHL1* genes and their mediation of the absorption and utilization of nitrogen and Pi under stress are worthy of further investigation.

Generally, *CsARR-B* genes were also expressed at relatively high levels in the roots (Figure 7), suggesting that this subfamily of TFs may be involved in root growth regulation. Salvi et al. (2020) indicated that, by interacting with auxin and PLETHORA, the ARR-B gene *ARR1* controls expansion of the division zone in *Arabidopsis* roots. Given that ARR-Bs serve as key regulators involved in cytokinin signal transduction, increasing evidence has shown that ARR-Bs also play important roles in the signaling of other phytohormones, such as auxin and zeatin (Safi et al., 2017; Xiang et al., 2021). Additionally, genes such as *CsAPRR1* were expressed at high levels in the stems, buds and flowers (Figure 8). In these tissues, the phytohormones cytokinin, auxin and zeatin accumulate at high levels and facilitate cell division and expansion. Moreover, the transcript levels of several of these genes, including *CsARR-B4c* and *CsARR-B4d*, were maintained at high levels among different tissues, implying that these genes may play fundamental roles in tea plant development and growth. In addition, we found that the transcript levels of several genes, such as *CsARR-B1b*, *CsAPRR1*, and *CsARR-B4c*, were significantly regulated in response to stress treatments, including cold, drought, salt and Al (Figures 10, 11, 12A). Correspondingly, it has been widely shown that *ARR-B* genes are upregulated in response to cold and heat stress (Peng et al., 2015; Xiang et al., 2021) and are considered key cold-responsive genes (Gong et al., 2018). Therefore, by modulating cytokinin, auxin and zeatin signaling, these differentially expressed *CsARR-B* genes might participate in the tea plant response to stress at some level.

*GLK* genes have been shown to function extensively in chloroplast development, photosynthesis regulation, the stress response, and fruit development (Fitter et al., 2002; Powell et al., 2012; Nadakuduti et al., 2014; Nagatoshi et al., 2016; Lupi et al., 2019). In particular, *GLK* genes were the first genes identified to control chloroplast structure, as defects in this gene resulted in pale green leaf blade color (Hall et al., 1998). In the present study, *CsGLK* genes were expressed at high levels in the leaves (Figure 8), which is associated with their functions in chloroplast and photosynthesis of leaves. Interestingly, we also found that the expression levels of *CsGLK1/2* genes were lower in white and yellow-green leaves than in green leaves (Figure 9). Numerous studies have demonstrated aberrant morphology of chloroplast ultrastructure and low chlorophyll contents in white, yellow, and yellow-green tea leaves. Recently, Hao et al. (2020) identified pale green branches with chloroplast defects from tea plant and found that *CsGLKs* were significantly downregulated in these mutant tissues. Unexpectedly, in the present study the expression levels of *CsBOA1* and *CsPCL1* were upregulated in both the white and the yellow-white leaves and downregulated in the green leaves (Figure 9). However, the functions of *BOA1* and *PCL1* are largely unknown in plants. Therefore, to some extent, we propose that the albinism and etiolation of tea plants mainly resulted from the downregulation of *CsGLKs*. In addition, we found that this subfamily of genes was regulated by stress treatments; this was especially the case for *CsBOA1*, which was significantly induced in response to Al treatment and drought (Figures 11, 12A), whereas it was downregulated in mature leaves in response to cold but upregulated in young leaves (Figure 10), implying that *CsGLK* genes are involved in tea plant stress responsiveness. Notably, Ahmad et al. (2019) showed that the GLK1/2-WRKY40 transcription module plays a negative regulatory role in the abscisic acid (ABA) response, indicating that *GLK* genes are also involved in ABA signaling. Overall, the current study provides insights into the diverse functions of *CsGARP* genes and lays a foundation for future research to determine the functions of *CsGARP* genes in tea plant.

## Data Availability Statement

The original contributions presented in this study are publicly available. This data can be found here: PRJNA843954.

## Author Contributions

CY conceived and designed the experiments and wrote the original draft. CY, QC, and JH performed the experiments. CL, JH, and QC analyzed the results. LL and LZ reviewed and edited the manuscript. All authors read and approved the final manuscript.

## Conflict of Interest

The authors declare that the research was conducted in the absence of any commercial or financial relationships that could be construed as a potential conflict of interest.

## Publisher’s Note

All claims expressed in this article are solely those of the authors and do not necessarily represent those of their affiliated organizations, or those of the publisher, the editors and the reviewers. Any product that may be evaluated in this article, or claim that may be made by its manufacturer, is not guaranteed or endorsed by the publisher.

## Footnotes

1
http://tpia.teaplant.org

2
http://kiwifruitgenome.org/organism/5

3
https://phytozome-next.jgi.doe.gov/pz/portal.html

4
https://www.arabidopsis.org/

5
http://evolgenius.info/#/

6
http://gsds.gao-lab.org/index.php

7
http://meme-suite.org/tools/meme

8
http://tpdb.shengxin.ren/

9
http://bioinformatics.psb.ugent.be/webtools/plantcare/html/

10
https://www.ncbi.nlm.nih.gov/tools/primer-blast/

## Supplementary Material

Supplementary Figure 1 Phylogenetic tree of GARP proteins from 19 species constructed by the ML method.

Supplementary Figure 2 Overview of multiple sequence alignment of 69 CsGARP proteins. The sequence alignment of 69 CsGARPs was conducted using ClustalX, and visual analysis was performed through Jalview.

Supplementary Figure 3 Chromosomal locations of 69 *CsGARP* genes.
