# Supplementary material for: Genome-Wide Identification and Characterization of GARP Transcription Factor Gene Family Members Reveal Their Diverse Functions in Tea Plant (Camellia sinensis)
Source: Front Plant Sci. 2022 Jun 30;13:947072. doi: 10.3389/fpls.2022.947072 (PMC9280663; doi:10.3389/fpls.2022.947072)
Supplement: Supplementary file 1 [file Table_1.DOCX]

**Supplementary Tables**

**Table S1** Summary of *GARP* TFs among 19 species

| Class | Order | Family | | Species | | Genome size (Mb) | The No. of GARP members in sub-families | | | | | Total No. |
| --- | --- | --- | --- | --- | --- | --- | --- | --- | --- | --- | --- | --- |
|  |  |  |  |  |  |  | KAN | NIGT1/HRS1  /HHO | PHR1/PHL1 | GLK | ARR-B |  |
| Angiosperms | Amborellales | | *Amborellaceae* | | *Amborella trichopoda* | 706.495 | 6 | 3 | 13 | 4 | 5 | 31 |
|  | Nymphaeales | | *Nymphaeaceae* | | *Nymphaea colorata* | 409 | 15 | 7 | 18 | 6 | 4 | 50 |
| Monocotyledoneae | Poales | | *Poaceae* | | *Oryza sativa* | 386.486 | 15 | 5 | 18 | 5 | 9 | 52 |
|  |  |  |  |  | *Sorghum bicolor* | 715.371 | 15 | 5 | 17 | 3 | 13 | 53 |
|  |  |  |  |  | *Zea mays L.* | 2192.4 | 21 | 6 | 16 | 3 | 10 | 56 |
|  |  |  | *Bromeliaceae* | | *Ananas comosus* | 382.056 | 11 | 2 | 10 | 4 | 7 | 34 |
| Superasterids | Solanales | | *Solanaceae* | | *Solanum tuberosum L.* | 760.236 | 13 | 4 | 15 | 8 | 20 | 60 |
|  |  |  |  |  | *Solanum lycopersicum* | 809.184 | 19 | 4 | 16 | 6 | 22 | 67 |
|  | Ericales | | *Actinidiaceae* | | *Actinidia chinensis* | 604.217 | 21 | 9 | 26 | 8 | 18 | 82 |
|  |  |  | *Theaceae* | | *Camellia sinensis* | 3105.37 | 17 | 10 | 21 | 9 | 12 | 69 |
|  | Gentianales | | *Rubiaceae* | | *Coffea canephora* | 1094.45 | 10 | 4 | 11 | 1 | 6 | 32 |
| Superrosids | Fabales | | *Fabaceae* | | *Cicer arietinum L.* | 511.684 | 10 | 4 | 16 | 7 | 14 | 51 |
|  |  |  |  |  | *Glycine max* | 994.355 | 30 | 15 | 34 | 10 | 28 | 117 |
|  | Brassicales | | *Brassicaceae* | | *Arabidopsis thaliana* | 119.75 | 9 | 8 | 16 | 9 | 14 | 56 |
|  | Myrtales | | *Myrtaceae* | | *Eucalyptus grandis* | 653.98 | 12 | 5 | 13 | 6 | 6 | 42 |
|  | Malpighiales | | *Salicaceae* | | *Populus trichocarpa* | 434.29 | 21 | 8 | 27 | 8 | 12 | 76 |
|  | Rosales | | *Rosaceae* | | *Prunus Persica* | 214.225 | 11 | 4 | 14 | 4 | 8 | 41 |
|  | Vitales | | *Vitaceae* | | *Vitis vinifera* | 427.211 | 11 | 4 | 16 | 4 | 11 | 46 |
|  | Malvales | | *Malvaceae* | | *Theobroma Cacao* | 335.437 | 10 | 4 | 16 | 7 | 11 | 48 |

**Table S2** Primers used for qRT–PCR detection and subcellular localization assay

| Name | Sequence |
| --- | --- |
| *qNIGT2e-F* | CAATGTCAAGGGCTCTGCAA |
| *qNIGT2e-R* | CCCTTTCAACCACCGGAGTC |
| *qNIGT2c-F* | ACCACCACCACTACGAGTCA |
| *qNIGT2c-R* | AGTCTGTATTTCTGGAGATGGCT |
| *qNIGT2f-F* | TGAGCTCTACCGATCAGAAACA |
| *qNIGT2f-R* | TCCGGCCACTGATAACTCCT |
| *qNIGT2g-F* | CTGCCCCTAACGGTCAATCA |
| *qNIGT2g-R* | GTGAACCCCCAAGTTGTTGC |
| *qNIGT2a-F* | TGTCCAAGCTCGACATTGCT |
| *qNIGT2a-R* | GAGAATCTTGCGGACCACCA |
| *qNIGT1c-F* | GCTAGAAGGTGTTGGTCCCC |
| *qNIGT1c-R* | TCTTCTTGAATGCAGCCGGT |
| *qNIGT1a-F* | AGTGGGGGTGCTTTTCATCC |
| *qNIGT1a-R* | TTTCTTAGTCGCAGTCGCCG |
| *qNIGT1b-F* | GCTAGAAGGTGTTGGTCCCC |
| *qNIGT1b-R* | ACAACATTCAAAGAAACGAACTGT |
| *qBOA1-F* | ATCTGACGCCGTTGTCTCAG |
| *qBOA1-R* | ACGCGGACAACGCATTAAAC |
| *qARR-B3b-F* | CCCAGCACAAAGTCTAGCCA |
| *qARR-B3b-R* | TTTGTTGTGGCTGTTGCGAG |
| *qKAN1d-F* | GTGGCCATGAAAGGGCTACT |
| *qKAN1d-R* | AGCTGCCCACTGTAGAGGTA |
| *qPHL6c-F* | CCACCCTGGAAAACCCCTCT |
| *qPHL6c-R* | CAGAAGCCTTGCTTTCTGGC |
| *qPHL6e-F* | TAGTGAACGAGCTACACCGA |
| *qPHL6e-R* | AGTAATCCCCATACTCGCTTTCA |
| *qGLK1-F* | GTATGGGCACCAACACCAAC |
| *qGLK1-R* | GAATGCCGGGGACCACTG |
| *qARR-B4c-F* | GTGCTTGATCAGGGACCACT |
| *qARR-B4c-R* | GCGTCATGCAAGCTTTTCCA |
| *qPHL6a-F* | TGCCAACACTGGAAGTGGAA |
| *qPHL6a-R* | GTTGAGAGTCGGGCAACTGA |
| *qPHL4a-F* | AGCACAACATCAGGTGCTCA |
| *qPHL4a-R* | GTGAAAAGGCCTTGCCATGT |
| *qPHL2a-F* | TATCCGTACGAGAGTGGGGT |
| *qPHL2a-R* | TGGGAGTTGCTTTGTCAGGG |
| *qPHL3a-F* | GGTGGACCTGACAAAGCAAC |
| *qPHL3a-R* | AGACGCTGCAATCCCATCTTT |
| *qAPL1-F* | AGGCTTGGAAAGCAACCTCA |
| *qAPL1-R* | ACTGTCATTCATACTACGTGCC |
| *CsNIGT1b-F* | CTTCTGCAGGGGCCCATGATCAGCGATAGTCACAATCA |
| *CsNIGT1b-R* | GGGAAATTCGAGCTCTTAATGAACATTGAAGATGACAAC |
| *CsKAN1b-F* | CTTCTGCAGGGGCCCATGAGAACTGGTTCGACAAC |
| *CsKAN1b-R* | GGGAAATTCGAGCTCTTAAAGAACTGTCAAGTCATTTGAT |
| *CsBOA1-F* | CTTCTGCAGGGGCCCATGGGGGAAGAAGTGAGGATG |
| *CsBOA1-R* | GGGAAATTCGAGCTCTTAGCTGTTTTGAAATTGAACG |

**Table S3** Gene duplication event analysis of *CsGARP* genes

| Gene_ID | GARP names | Duplicate type |
| --- | --- | --- |
| CSS0000687.1 | *CsGLK1* | Dispersed |
| CSS0001412.1 | *CsKAN1f* | Dispersed |
| CSS0003719.1 | *CsBOA1* | Dispersed |
| CSS0006502.1 | *CsKAN2f* | Dispersed |
| CSS0008911.1 | *CsPHL4c* | Dispersed |
| CSS0010394.1 | *CsARR-B4d* | Dispersed |
| CSS0017468.1 | *CsARR-B2b* | Dispersed |
| CSS0023934.1 | *CsKAN1e* | Dispersed |
| CSS0023963.1 | *CsPHL3a* | Dispersed |
| CSS0027631.1 | *CsPHL3b* | Dispersed |
| CSS0031273.1 | *CsNIGT2b* | Dispersed |
| CSS0031744.1 | *CsKAN2e* | Dispersed |
| CSS0034150.1 | *CsPCL1* | Dispersed |
| CSS0035819.1 | *CsPHL6d* | Dispersed |
| CSS0036129.1 | *CsMYR1* | Dispersed |
| CSS0037714.2 | *CsAPRR1* | Dispersed |
| CSS0038558.1 | *CsPHL4b* | Dispersed |
| CSS0041137.1 | *CsMTF1* | Dispersed |
| CSS0041856.1 | *CsPHL2b* | Dispersed |
| CSS0043810.1 | *CsARR-B4b* | Dispersed |
| CSS0044591.1 | *CsNIGT2d* | Dispersed |
| CSS0001889.1 | *CsGLK2* | Proximal |
| CSS0005408.1 | *CsPHL5b* | Proximal |
| CSS0007601.1 | *CsAPL1* | Proximal |
| CSS0017966.1 | *CsPHL5a* | Proximal |
| CSS0018792.1 | *CsAPL2* | Proximal |
| CSS0031258.1 | *CsNIGT1c* | Proximal |
| CSS0036016.1 | *CsGLK3* | Proximal |
| CSS0049732.1 | *CsKAN2g* | Proximal |
| CSS0003450.1 | *CsKAN2c* | WGD or Segmental |
| CSS0004133.1 | *CsKAN1c* | WGD or Segmental |
| CSS0006078.1 | *CsNIGT1b* | WGD or Segmental |
| CSS0006111.1 | *CsPHL2a* | WGD or Segmental |
| CSS0006880.1 | *CsKAN1a* | WGD or Segmental |
| CSS0007741.1 | *CsNIGT2a* | WGD or Segmental |
| CSS0007827.1 | *CsNIGT2c* | WGD or Segmental |
| CSS0008648.1 | *CsARR-B1b* | WGD or Segmental |
| CSS0011420.1 | *CsKAN2i* | WGD or Segmental |
| CSS0013426.1 | *CsARR-B2d* | WGD or Segmental |
| CSS0014748.1 | *CsNIGT2g* | WGD or Segmental |
| CSS0015223.1 | *CsPHL6a* | WGD or Segmental |
| CSS0018275.1 | *CsKAN2d* | WGD or Segmental |
| CSS0018618.1 | *CsPHL1a* | WGD or Segmental |
| CSS0019219.1 | *CsPHL4a* | WGD or Segmental |
| CSS0019363.1 | *CsPHL6b* | WGD or Segmental |
| CSS0021269.1 | *CsKAN1h* | WGD or Segmental |
| CSS0023102.1 | *CsKAN1b* | WGD or Segmental |
| CSS0023343.1 | *CsPHL2c* | WGD or Segmental |
| CSS0024071.1 | *CsARR-B4a* | WGD or Segmental |
| CSS0024543.1 | *CsARR-B3a* | WGD or Segmental |
| CSS0026556.1 | *CsMTF3* | WGD or Segmental |
| CSS0026971.1 | *CsPHL6c* | WGD or Segmental |
| CSS0031217.1 | *CsNIGT1a* | WGD or Segmental |
| CSS0035353.1 | *CsARR-B1a* | WGD or Segmental |
| CSS0037737.1 | *CsARR-B2a* | WGD or Segmental |
| CSS0039772.1 | *CsPHL6e* | WGD or Segmental |
| CSS0039789.1 | *CsARR-B2c* | WGD or Segmental |
| CSS0039972.1 | *CsKAN1d* | WGD or Segmental |
| CSS0042562.1 | *CsARR-B4c* | WGD or Segmental |
| CSS0042630.3 | *CsPHL6f* | WGD or Segmental |
| CSS0043198.1 | *CsKAN2h* | WGD or Segmental |
| CSS0043572.1 | *CsKAN2b* | WGD or Segmental |
| CSS0044249.1 | *CsMTF2* | WGD or Segmental |
| CSS0044539.1 | *CsNIGT2e* | WGD or Segmental |
| CSS0045704.1 | *CsPHL1b* | WGD or Segmental |
| CSS0047374.1 | *CsKAN1g* | WGD or Segmental |
| CSS0047937.1 | *CsKAN2a* | WGD or Segmental |
| CSS0049001.1 | *CsNIGT2f* | WGD or Segmental |
| CSS0049738.1 | *CsARR-B3b* | WGD or Segmental |

**Table S4** Parameters and dates of duplication events of the *CsGARP* gene pairs

| Seq_1 | Seq_2 | Ka | Ks | Ka_Ks | Effective  Length | Average  S-sites | Average  N-sites | cN | cS | pN | pS | Diverged years (MYA) | Positive selection |
| --- | --- | --- | --- | --- | --- | --- | --- | --- | --- | --- | --- | --- | --- |
| *CsKAN1c* | *CsKAN1d* | 0.14 | 0.47 | 0.31 | 1065 | 240.75 | 824.25 | 108.50 | 84.50 | 0.13 | 0.35 | 36.40 | NO |
| *CsARR-B1a* | *CsARR-B1b* | 0.00 | 0.01 | 0.26 | 849 | 177.17 | 671.83 | 1.00 | 1.00 | 0.00 | 0.01 | 0.44 | NO |
| *CsARR-B2a* | *CsARR-B2c* | 0.12 | 0.39 | 0.32 | 1710 | 375.83 | 1334.17 | 153.58 | 114.42 | 0.12 | 0.30 | 30.04 | NO |
| *CsARR-B2a* | *CsARR-B2d* | 0.12 | 0.39 | 0.32 | 1710 | 375.83 | 1334.17 | 153.58 | 114.42 | 0.12 | 0.30 | 30.04 | NO |
| *CsARR-B3a* | *CsARR-B3b* | 0.52 | 1.82 | 0.29 | 1182 | 257.42 | 924.58 | 348.92 | 176.08 | 0.38 | 0.68 | 140.22 | NO |
| *CsARR-B4a* | *CsARR-B4c* | 0.09 | 0.34 | 0.28 | 1998 | 458.42 | 1539.58 | 136.08 | 124.92 | 0.09 | 0.27 | 26.04 | NO |
| *CsKAN1a* | *CsKAN1b* | 0.16 | 0.37 | 0.42 | 861 | 192.83 | 668.17 | 94.67 | 56.33 | 0.14 | 0.29 | 28.47 | NO |
| *CsKAN2h* | *CsKAN2i* | 0.18 | 0.50 | 0.35 | 405 | 85.33 | 319.67 | 50.67 | 31.33 | 0.16 | 0.37 | 38.79 | NO |
| *CsMTF2* | *CsMTF3* | 0.03 | 0.05 | 0.60 | 819 | 195.50 | 623.50 | 17.17 | 8.83 | 0.03 | 0.05 | 3.58 | NO |
| *CsNIGT1a* | *CsNIGT1b* | 0.28 | 0.80 | 0.35 | 732 | 154.25 | 577.75 | 135.33 | 75.67 | 0.23 | 0.49 | 61.23 | NO |
| *CsNIGT2a* | *CsNIGT2c* | 0.13 | 0.55 | 0.23 | 1359 | 315.42 | 1043.58 | 120.67 | 122.33 | 0.12 | 0.39 | 41.99 | NO |
| *CsNIGT2e* | *CsNIGT2f* | 0.49 | 1.92 | 0.26 | 852 | 190.42 | 661.58 | 239.17 | 131.83 | 0.36 | 0.69 | 147.98 | NO |
| *CsNIGT2e* | *CsNIGT2g* | 0.47 | 1.55 | 0.30 | 840 | 185.17 | 654.83 | 228.67 | 121.33 | 0.35 | 0.66 | 119.34 | NO |
| *CsNIGT2f* | *CsNIGT2g* | 0.20 | 0.57 | 0.35 | 1119 | 246.42 | 872.58 | 153.00 | 98.00 | 0.18 | 0.40 | 43.58 | NO |
| *CsPHL1a* | *CsPHL1b* | 0.19 | 0.63 | 0.30 | 897 | 193.83 | 703.17 | 119.17 | 82.83 | 0.17 | 0.43 | 48.65 | NO |
| *CsPHL2a* | *CsPHL2c* | 0.10 | 0.36 | 0.29 | 594 | 127.58 | 466.42 | 45.50 | 36.50 | 0.10 | 0.29 | 27.71 | NO |
| *CsPHL6a* | *CsPHL6b* | 0.02 | 0.04 | 0.57 | 1374 | 312.42 | 1061.58 | 25.17 | 12.83 | 0.02 | 0.04 | 3.25 | NO |
| *CsPHL6a* | *CsPHL6c* | 0.18 | 0.33 | 0.55 | 1392 | 320.00 | 1072.00 | 169.58 | 84.42 | 0.16 | 0.26 | 25.00 | NO |
| *CsPHL6c* | *CsPHL6e* | 0.39 | 1.45 | 0.27 | 1371 | 310.42 | 1060.58 | 322.83 | 199.17 | 0.30 | 0.64 | 111.57 | NO |
| *CsPHL6d* | *CsPHL6f* | 0.32 | 1.23 | 0.26 | 1005 | 222.42 | 782.58 | 203.50 | 134.50 | 0.26 | 0.60 | 94.68 | NO |
