# Supplementary figures and images for: Genome-Wide Identification and Characterization of GARP Transcription Factor Gene Family Members Reveal Their Diverse Functions in Tea Plant (Camellia sinensis)
Source: Front Plant Sci. 2022 Jun 30;13:947072. doi: 10.3389/fpls.2022.947072 (PMC9280663; doi:10.3389/fpls.2022.947072)

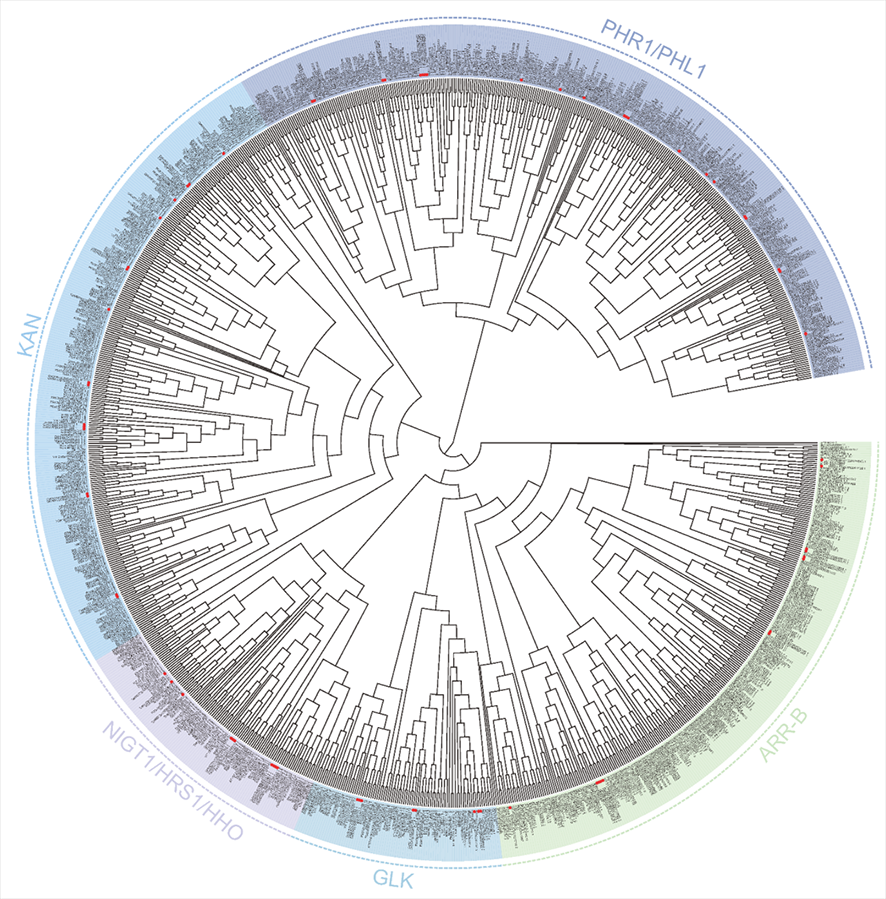

Supplement: Supplementary Figure 1 — Phylogenetic tree of GARP proteins from 19 species constructed by the ML method. [file Image_1.TIF]

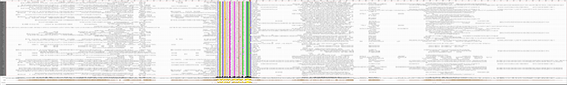

Supplement: Supplementary Figure 2 — Overview of multiple sequence alignment of 69 CsGARP proteins. The sequence alignment of 69 CsGARPs was conducted using ClustalX, and visual analysis was performed through Jalview. [file Image_2.TIF]

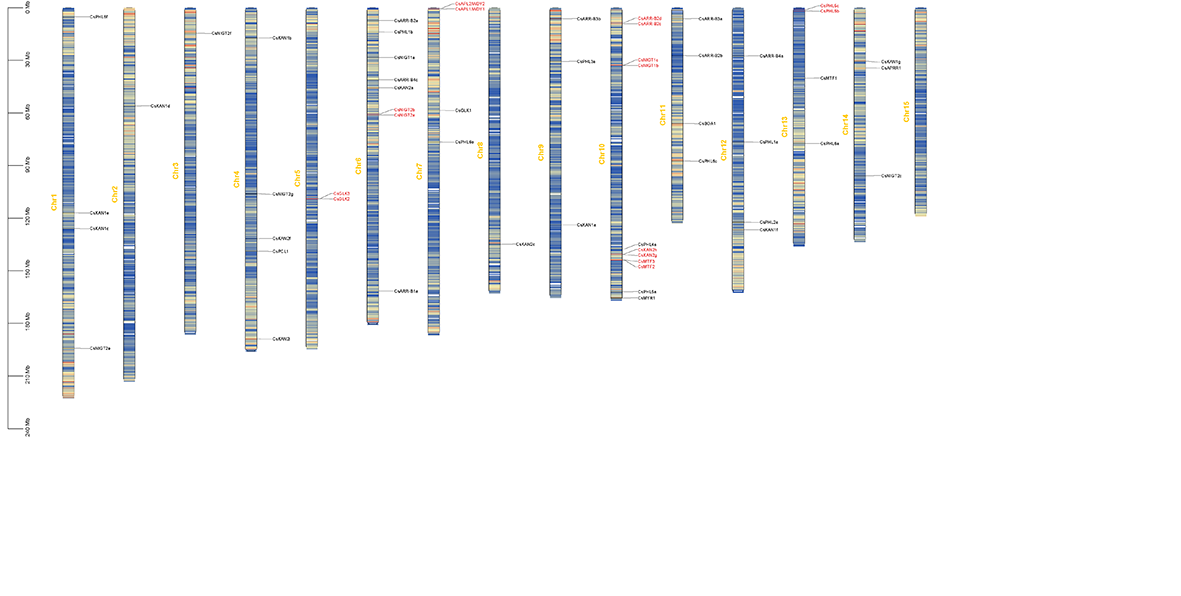

Supplement: Supplementary Figure 3 — Chromosomal locations of 69 CsGARP genes. [file Image_3.TIFF]
